# Structural insights into reptarenavirus cap-snatching machinery

**DOI:** 10.1371/journal.ppat.1006400

**Published:** 2017-05-15

**Authors:** Maria Rosenthal, Nadja Gogrefe, Dominik Vogel, Juan Reguera, Bianka Rauschenberger, Stephen Cusack, Stephan Günther, Sophia Reindl

**Affiliations:** 1 Department of Virology, Bernhard-Nocht-Institute for Tropical Medicine, Hamburg, Germany; 2 Aix-Marseille Université, INSERM, CNRS, AFMB UMR 7257, Marseille, France; 3 European Molecular Biology Laboratory, Grenoble Outstation, Grenoble, France; Harvard Medical School, UNITED STATES

## Abstract

Cap-snatching was first discovered in influenza virus. Structures of the involved domains of the influenza virus polymerase, namely the endonuclease in the PA subunit and the cap-binding domain in the PB2 subunit, have been solved. Cap-snatching endonucleases have also been demonstrated at the very N-terminus of the L proteins of mammarena-, orthobunya-, and hantaviruses. However, a cap-binding domain has not been identified in an arena- or bunyavirus L protein so far. We solved the structure of the 326 C-terminal residues of the L protein of California Academy of Sciences virus (CASV), a reptarenavirus, by X-ray crystallography. The individual domains of this 37-kDa fragment (L-Cterm) as well as the domain arrangement are structurally similar to the cap-binding and adjacent domains of influenza virus polymerase PB2 subunit, despite the absence of sequence homology, suggesting a common evolutionary origin. This enabled identification of a region in CASV L-Cterm with similarity to a cap-binding site; however, the typical sandwich of two aromatic residues was missing. Consistent with this, cap-binding to CASV L-Cterm could not be detected biochemically. In addition, we solved the crystal structure of the corresponding endonuclease in the N-terminus of CASV L protein. It shows a typical endonuclease fold with an active site configuration that is essentially identical to that of known mammarenavirus endonuclease structures. In conclusion, we provide evidence for a presumably functional cap-snatching endonuclease in the N-terminus and a degenerate cap-binding domain in the C-terminus of a reptarenavirus L protein. Implications of these findings for the cap-snatching mechanism in arenaviruses are discussed.

## Author summary

Arenaviruses occur worldwide and can cause severe, often fatal hemorrhagic fever in humans. Vaccines and effective treatments are not available. Arenaviruses replicate in the cytoplasm of infected cells and since they cannot synthesize cap-structures they use a mechanism called cap-snatching to steal cap structures from host mRNAs for viral transcription. This mechanism is an attractive drug target, as it is essential for virus replication and virus specific. However, the arenaviral components of this mechanism are poorly defined compared to influenza virus, the prototypic cap-snatching virus. We present the first crystal structures of two putative components of the California Academy of Sciences arenavirus cap-snatching machinery, namely the isolated N- and C-termini of the viral RNA polymerase (L protein). The N-terminus harbors what looks like a functional cap-snatching endonuclease. The L protein C-terminus, despite complete sequence divergence, shows overall structural similarity to the C-terminal region of influenza virus polymerase PB2 subunit, suggesting a common evolutionary origin. A domain clearly related to the PB2 cap-binding domain is present, although cap-binding could not be biochemically demonstrated. The determined structures provide the basis for future research to unravel the details of the arenavirus cap-snatching mechanism and its potential as a target for drug development.

## Introduction

The family of arenaviruses is divided in two genera: mammarenaviruses and reptarenaviruses. With the notable exception of Tacaribe virus, rodents are described as the natural reservoirs for mammarenaviruses. Reptarenaviruses have only been found in captive snakes [1]. Some arenaviruses such as Lassa virus (LASV), Junin virus and Machupo virus, can cause severe human disease with hemorrhagic and neurological symptoms. To date, the only drug available for treatment of arenavirus infections is ribavirin, which presumably targets viral replication [2].

Arenaviruses are enveloped particles that contain two single stranded negative sense RNA segments. The two genome segments code for four viral proteins, the nucleoprotein (NP), the glycoprotein-precursor, the small matrix protein Z and the large > 200 kDa L protein which harbors the viral RNA-dependent RNA polymerase. The minimal viral components for genome replication and transcription are the viral RNA, NP, and the L protein [3]. The L protein synthesizes two distinct RNA species: (i) the antigenomic and genomic RNA as products of genome replication and (ii) the shorter capped viral mRNAs during transcription. To initiate viral transcription, the L protein presumably uses a process called cap-snatching. It is assumed that the L protein cleaves host cell mRNAs downstream of the 5'-cap structure and uses this short capped RNA as a primer for viral mRNA synthesis. Consistent with this hypothesis 4–5 non-templated nucleotides are found at the 5'-ends of viral mRNAs and there is an endonuclease in the N-terminal region of the L protein [4–7]. The prototype of cap-snatching viruses is influenza virus [8], which harbors an endonuclease in the PA subunit of the viral polymerase as well as a cap-binding site in the PB2 subunit [9–11]. Given the phylogenetic relatedness and similarities in the replication cycle of orthomyxoviruses and arenaviruses—both are segmented negative strand RNA viruses—it is reasonable to assume that the arenavirus L protein harbors a cap-binding site as well, although there is no direct evidence for this [12]. Previous functional data obtained with a LASV replicon system suggested that the cap-binding site might be located in the C-terminus of the L protein [13].

To further characterize the cap-snatching machinery of arenaviruses, we attempted to solve the structure of N- and C-terminal domains of L proteins of various arenaviruses. Eventually, we have been successful with the L protein of the California Academy of Sciences virus (CASV), which is a reptarenavirus. Here we present the crystal structures of the two terminal domains of the CASV L protein: the cap-snatching endonuclease in the N-terminus and the 326 C-terminal residues, which, by analogy to LASV, might play a role in transcription [13]. The active site of the endonuclease is nearly identical to other related enzymes, suggesting that reptarenaviruses use a cap-snatching mechanism for mRNA synthesis. The C-terminal domain is structurally related to the influenza virus PB2 protein and features a putative non-functional cap-binding site. We speculate about its role in the cap-snatching mechanism of arenaviruses and discuss our data in the context of available structural and functional data from other segmented negative strand RNA viruses.

## Results

### Construct design and solubility screening for C-terminal fragments of the arenavirus L protein

To obtain soluble protein fragments of the C-terminal domain, we cloned and tested more than 120 different L protein fragments from 20 arenavirus species covering a wide phylogenetic spectrum for soluble expression in *Escherichia coli* (see S3 Table). Fifteen percent of the proteins were initially soluble. Soluble candidates were purified by nickel affinity and size exclusion chromatography and tested for stability. About five percent of the fragments were monodisperse and stable and used for crystallization trials. Optimization of expressed fragments using bioinformatics, limited proteolysis, and thermal stability assays led to the C-terminal 326 amino acids of the CASV L protein (residues 1721–2046; residue numbering refers to the full-length L protein) with N-terminal His-tag as best candidate for structure determination.

### Structure of CASV L protein C-terminus

**Fig 1:**
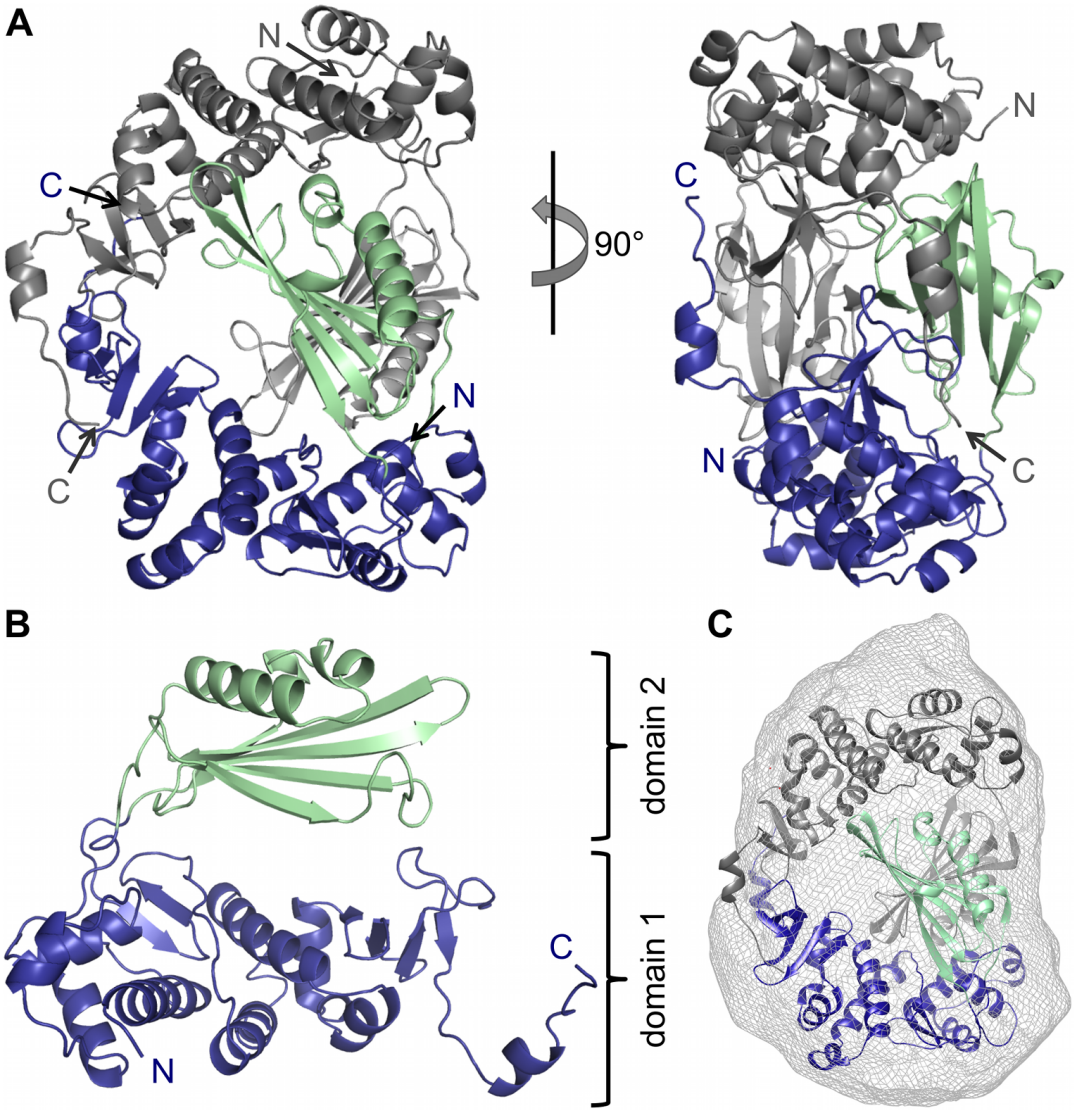
Atomic structure of CASV L-Cterm. **A)** The structure of the protein dimer in the asymmetric unit is shown as a ribbon diagram in front and side view. Chain A is colored in blue and green, chain B is colored in dark and light grey. N- and C-termini are labelled. **B)** Chain A is shown as a ribbon diagram. N- and C-termini are labelled. Domain 1 is shown in blue, domain 2 in green. **C)** Superimposition of SAXS-derived molecular shape with the crystal structure (ribbon diagram) confirms the dimeric conformation of the protein at 1 mg/ml in solution.

After His-tag cleavage, the purified seleno-methionine-labelled protein was successfully crystallized and the structure was solved using the single anomalous dispersion method. The protein (called CASV L-Cterm) crystallized in space group P2_1_2_1_2_1_ with two molecules per asymmetric unit and the structure could be refined to a resolution of 2 Å (Fig 1A and 1B, S1 Table). Except for residues 1748, 1762 and 1768 in chain A and the region comprising residues 2034–2040 in chain B, clear electron density was observed for the structure. The protein crystallized as a dimer, which is not fully symmetric. The only notable difference between the monomers lies in the flexible loops connecting the two domains described below. This dimeric form is also observed in solution as revealed by size-exclusion chromatography and SAXS measurements (Fig 1C, S1A Fig). The protein monomer is U-shaped and consists of two separate domains, (i) a mainly α-helical domain (domain 1) composed of residues 1721–1793 and 1894–2046 with a long C-terminal tail and (ii) a domain (domain 2) consisting of a large β-sheet as well as one long and two short α-helices (residues 1794–1894) (Fig 1B, blue and green respectively). The second domain is inserted into the sequence of the first one and both domains are connected by two long flexible linkers with barely any additional contacts.

In the crystallized dimer the two U-shaped monomers interlock with each other to form a ring with a hole in the middle with a buried surface area of approximately 3000 Å^2^ between the monomers. The most intensive intermolecular contacts are between the very C-terminal 40 residues of each chain (buried surface area 1100 Å^2^).

### Structure-based similarity search to identify possible functions

To identify known structural homologs of our structure we used the DALI program for protein structure comparison [14] and performed the search with the whole monomer and with the two domains separately. For the mainly α-helical domain 1, no meaningful hit could be identified. The results included a variety of proteins such as exportins, importins, protein phosphatases, cytoskeleton-associated proteins, glutathione S-transferase as well as the eIF4G subunit of eukaryotic translation initiation factor 4F. All these hits had very low Z-scores (< 4.6) and no convincing structural similarity to L-Cterm.

Interestingly, for L-Cterm domain 2 the list contained the cap-binding domain of influenza virus PB2, which was also found when using the full monomer of CASV L-Cterm as search model. Other hits for domain 2 were acetyltransferases, sulfatases, methyltransferases, β-lactamases, and TATA-box binding proteins, again with relatively low Z-scores (< 5.0).

### Structural similarities between CASV L-Cterm and influenza virus PB2

**Fig 2:**
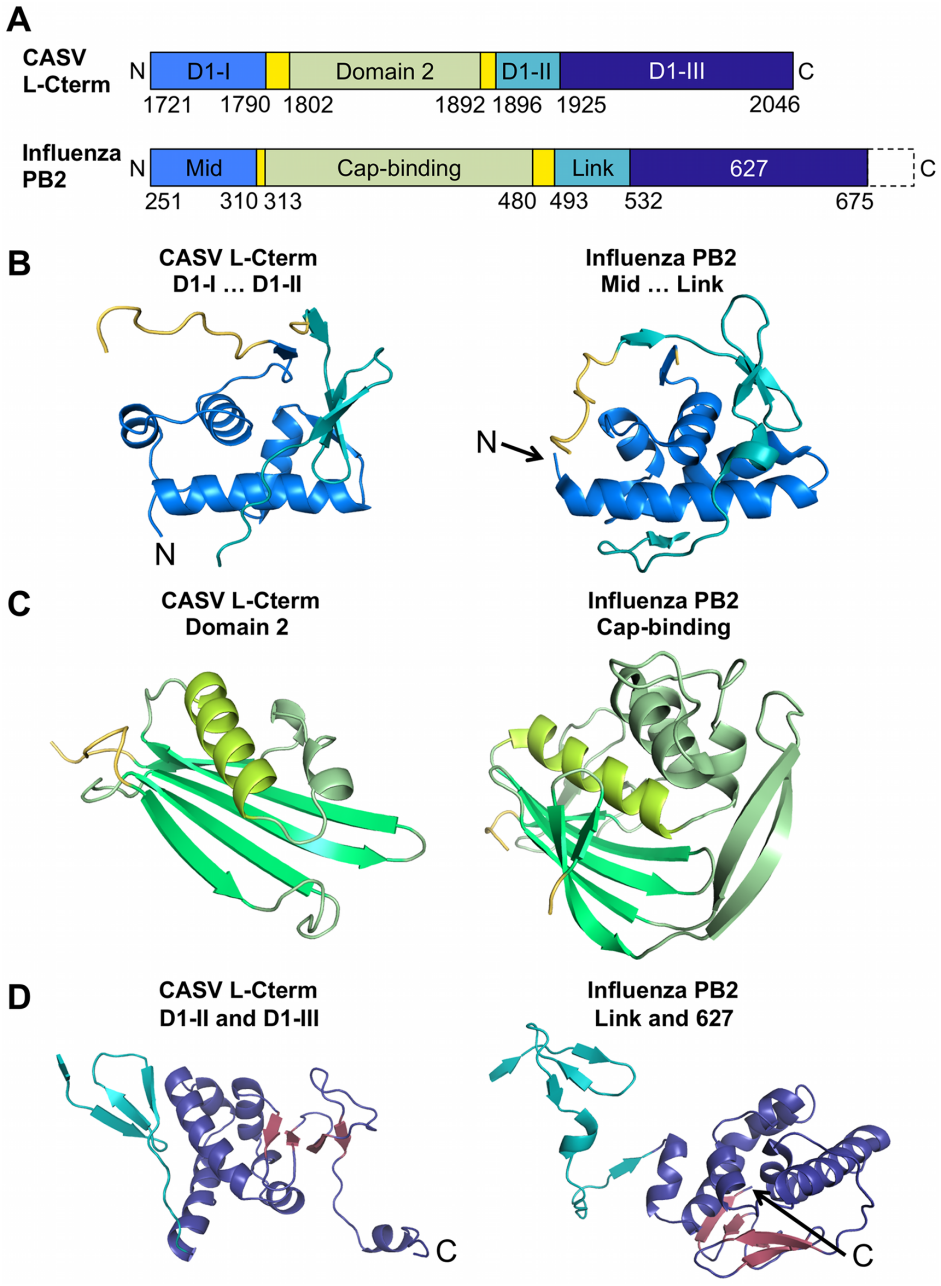
Comparison of CASV L-Cterm structure with influenza PB2 (PDB ID 5FMM) structure. **A)** Comparison of domain arrangements within PB2 and L-Cterm. Identifiers of the areas within the protein are shown in the bars. Domain 1 (D1) of L-Cterm is separated into three parts (D1-I, D1-II, and D1-III). Linkers to domain 2 or the cap-binding domain are colored in yellow. Residue numbers of the differently colored areas are given below the bars. N- and C-termini are labelled. C-terminal parts of PB2 missing in the figure are indicated by dashed lines. **B)** Structures of parts I and II of L-Cterm domain 1 (left panel) and the mid-link domains of PB2 (right panel) are shown as ribbon diagrams. Colors are coded as presented in A). Linkers to domain 2 and the cap-binding domain are shown in yellow. N-termini are labelled. **C)** Comparison of L-Cterm domain 2 (left panel) with PB2 cap-binding domain (right panel) with structures presented as ribbon diagrams. Structurally similar elements have similar colors. Linkers to other domains are colored in yellow. **D)** Structural comparison of parts II and III of L-Cterm domain 1 (left panel) and link-627 domains of PB2 (right panel). Structures are shown as ribbon diagrams. Colors are coded as in A). β-strands of part III of L-Cterm domain 1 and 627-domain of PB2 are colored in red. C-termini are labelled.

**Fig 3:**
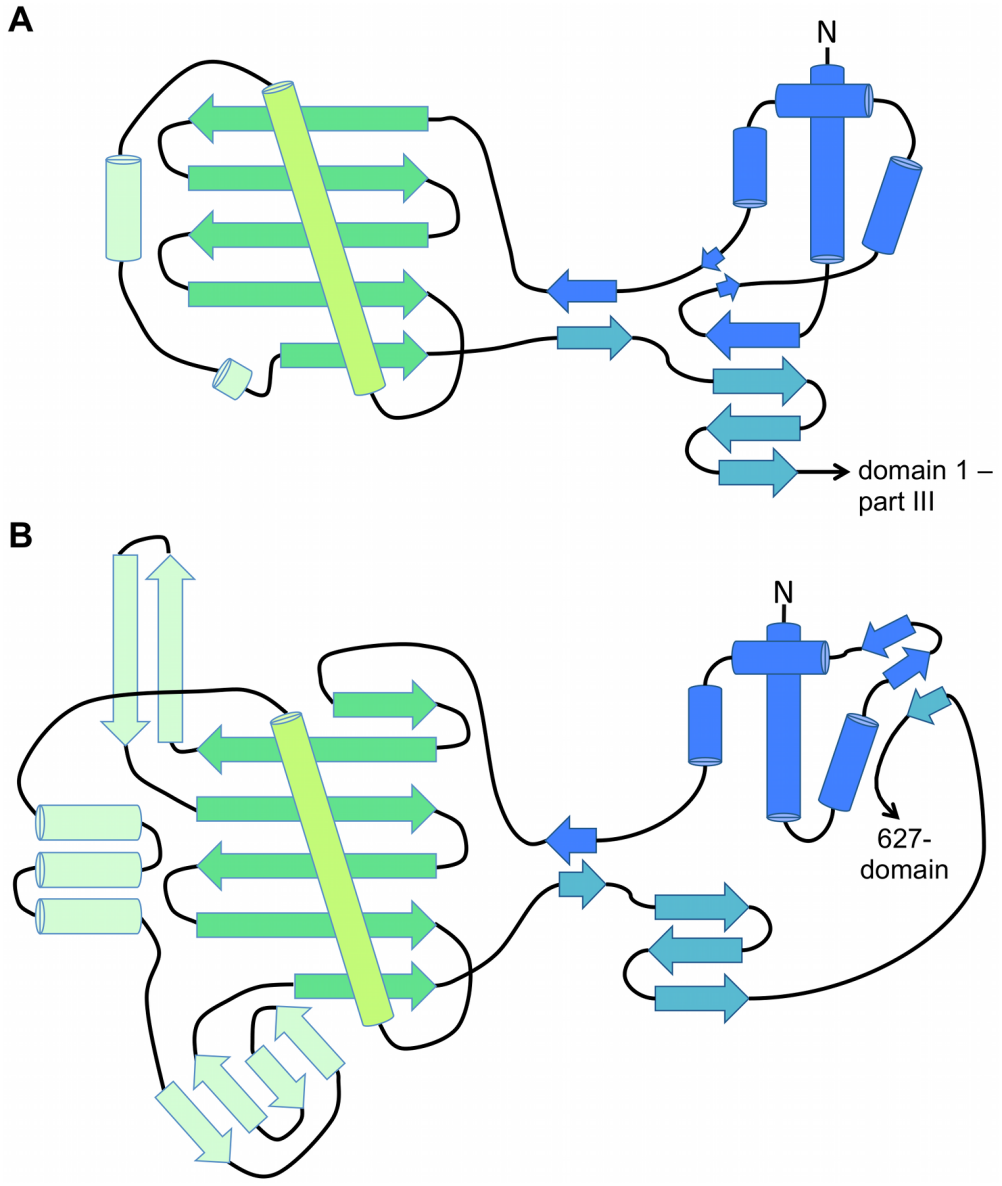
Topological overview of CASV L-Cterm and influenza virus PB2 structures. A schematic representation of the general domain architecture of **A)** CASV L-Cterm domain 1 –parts I and II (colored in blue and teal, respectively) and domain 2 (colored in greens) as well as of **B)** influenza virus PB2 mid-domain (blue), cap-binding-domain (greens) and linker-domain (teal). α-Helices are shown as cylinders, β-strands as large arrows. The N-termini are labelled and the protein parts that follow the represented domains are indicated by a small arrow with names given.

Despite a complete lack of sequence homology CASV L-Cterm and influenza PB2 show a remarkable similarity in overall domain architecture and sub-domain topology (Figs 2 and 3, influenza virus PB2 domains are drawn according to structure from ref. [15]). First, part I of CASV L-Cterm domain 1 (residues 1721–1790) is similar to the mid-domain of influenza virus PB2. Both are composed of four α-helices that are followed by a loop connecting with L-Cterm domain 2 or the PB2 cap-binding domain, respectively (Figs 2B and 3). Second, L-Cterm domain 1 part II (residues 1896–1924) is similar to the link region of PB2; both comprise a three-stranded β-sheet (Figs 2B, 2D and 3). Third, L-Cterm domain 1 part III (residues 1925–2046) corresponds to PB2 627-domain. Both regions comprise an α-helical bundle followed by a four-stranded small β-sheet, albeit in different orientations (Fig 2D). Only the acidic C-terminal tail of CASV L-Cterm (see also S2 and S10 Figs) is absent in influenza, which instead has a small domain containing the terminal nuclear localization sequence.

**Fig 4:**
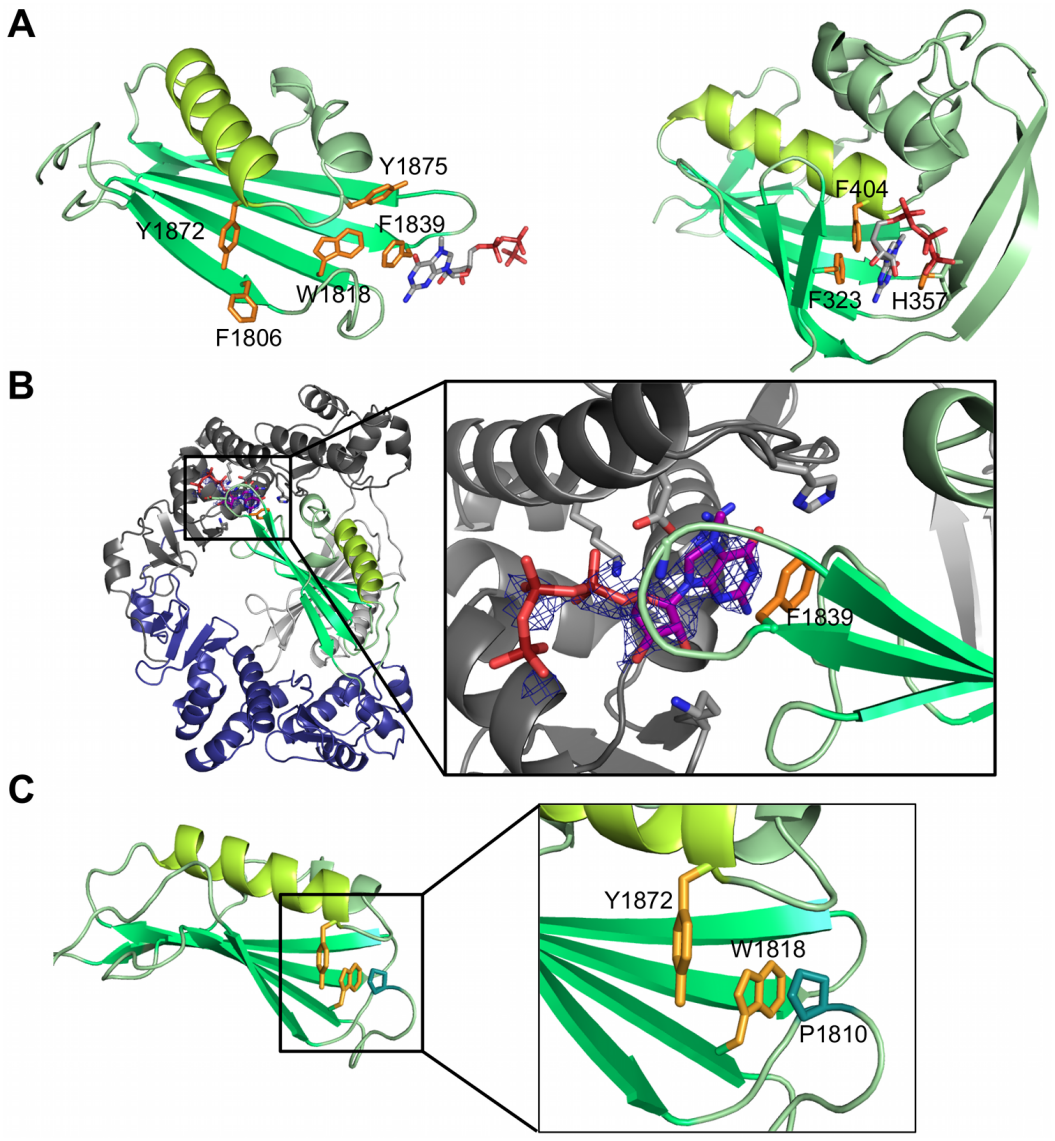
Examination of CASV L-Cterm cap-binding in comparison to influenza virus PB2. **A)** Comparison of CASV L-Cterm domain 2 (left panel) with PB2 cap-binding domain (PDB ID 2VQZ, right panel) with structures presented as ribbon diagrams. Structurally similar elements have the same color. Potential cap-binding aromatic residues in CASV L-Cterm and actual cap-binding residues in PB2 are shown as sticks and colored in orange. Bound m^7^GTP molecules are shown as sticks. **B)** The figure shows binding of m^7^GTP to the CASV L-Cterm dimer in the crystal after soaking experiments. An overview (left) and close-up (right) are shown. CASV L-Cterm is presented as a ribbon diagram with the residues relevant for binding shown as sticks. m^7^GTP is shown as sticks and the surrounding electron density (2|Fo|-|Fc| map at 2σ) as blue mesh. **C)** Interaction of W1818 with P1810 from a neighboring loop. CASV L-Cterm domain 2 is shown as green ribbon diagram, potential cap-binding residue Y1872 and W1818 are shown as orange sticks, and P1810 as blue sticks.

Most importantly, the highest degree of similarity was seen between the L-Cterm domain 2 and the PB2 cap-binding domain (Fig 2C). Both are formed by an antiparallel β-sheet packed against 3–4 α-helices. PB2 has a β-hairpin structure inserted between two strands of the β-sheet, which is lacking in domain 2 of L-Cterm. The latter features only a long loop at the homologous position (Figs 2C and 3). In PB2, the cap is bound in between F404 protruding from the end of the long helix (Fig 4A, right panel, helix shown in light green) and H357 located in the β-hairpin. Domain 2 of L-Cterm also contains an aromatic residue (Y1872) at the end of the homologous long helix (Fig 4A, left panel) pointing in the same direction as the F404 in PB2. As the β-hairpin is absent in the CASV L-Cterm, there is no homologue for the histidine residue. A possible candidate in L-Cterm to form an aromatic sandwich as seen in PB2 [9] could be W1818 that protrudes from the second β-strand. However, this residue is not in a conformation to form an aromatic sandwich as seen in PB2. The hypothetical conformational changes needed for W1818 side chain to get engaged in such an interaction are not possible in our structure, as P1810 from a neighboring loop tightly interacts with W1818 and holds the loop and thus the side chain of W1818 in place (Fig 4C). In conclusion, L-Cterm domain 2 is structurally similar to the PB2 cap-binding domain, although the typical aromatic sandwich for cap-binding is not complete.

Besides the structural organization of the isolated domains, their arrangement in the primary structure is conserved between influenza virus and CASV (Figs 2A and 3): in both PB2 and L-Cterm the cap-binding domain and domain 2, respectively, are inserted in the polypeptide chain at similar positions via two flexible linkers.

### Cap-binding studies with CASV L-Cterm

To test whether the CASV L-Cterm might bind to cap-structures despite an unfavorable arrangement of the aromatic residues in the crystal, we conducted several experiments using the cap-analogue m^7^GTP. First, the cap-analogue was soaked into the CASV L-Cterm crystals. However, electron density did not appear in the cavity formed by Y1872, F1806, and W1818, i.e. in the position expected by comparison to PB2 (Fig 4A and 4B). Instead, the cap-analogue was bound to F1839 at the periphery of the β-sheet in between the two CASV L-Cterm monomers. There was no second aromatic residue found in any symmetry related molecule suggesting m^7^GTP was not bound by an authentic cap-binding site. In fact the observed electron density was neither strong nor covering the full m^7^GTP molecule (Fig 4B). As mentioned, the dimeric form of the protein in the crystal is not fully symmetric and we found the m^7^GTP only bound between domain 2 of chain A and domain 1 of chain B, where the interface is slightly more open compared to the interface between domain 2 of chain B and domain 1 of chain A. We also tested the cap-binding ability of CASV L-Cterm in m^7^GTP-agarose pull-down assays. Whereas PB2 and eukaryotic initiation factor 4E (eIF4E), a eukaryotic cap-binding protein, bound to m^7^GTP-agarose, we could not detect binding of CASV L-Cterm (S6A Fig). Additionally, we could not observe an effect of m^7^GTP on the thermal stability of CASV L-Cterm or binding of CASV L-Cterm to capped RNA in a radioactive gel shift assay (S7 and S6B Figs).

### Role of protein dimerization and domain 1 for the cap-binding function

The dimer formation observed for CASV L-Cterm both in solution and in the crystal is presumably an artifact due to expression of the isolated C-terminal fragment of the L protein and not existent in the context of the full-length L protein. As the putative cap-binding site is close to the dimer interface, we tested whether the presence of L-Cterm domain 1 and/or the dimerization of CASV L-Cterm may prevent the protein from binding to m^7^GTP by locking the protein in a non-natural conformation. To this end, we attempted to block dimerization of L-Cterm. We analyzed the dimer interface and designed a mutant protein in which the C-terminal 14 residues are lacking (deltaC). These mostly negatively charged residues interact with a positively charged patch on the second molecule (S10 Fig), forming one third of the dimer interface. The deltaC construct was indeed purely monomeric according to SAXS measurements (S1C Fig), however, it did not bind to m^7^GTP-agarose (S6A Fig) and was not thermally stabilized by m^7^GTP (S7 Fig). Although weak binding to RNA was observed in gel shift assays, this affinity was not cap-specific (S6B and S6C Fig). Therefore, no further experiments were conducted with this fragment.

**Fig 5:**
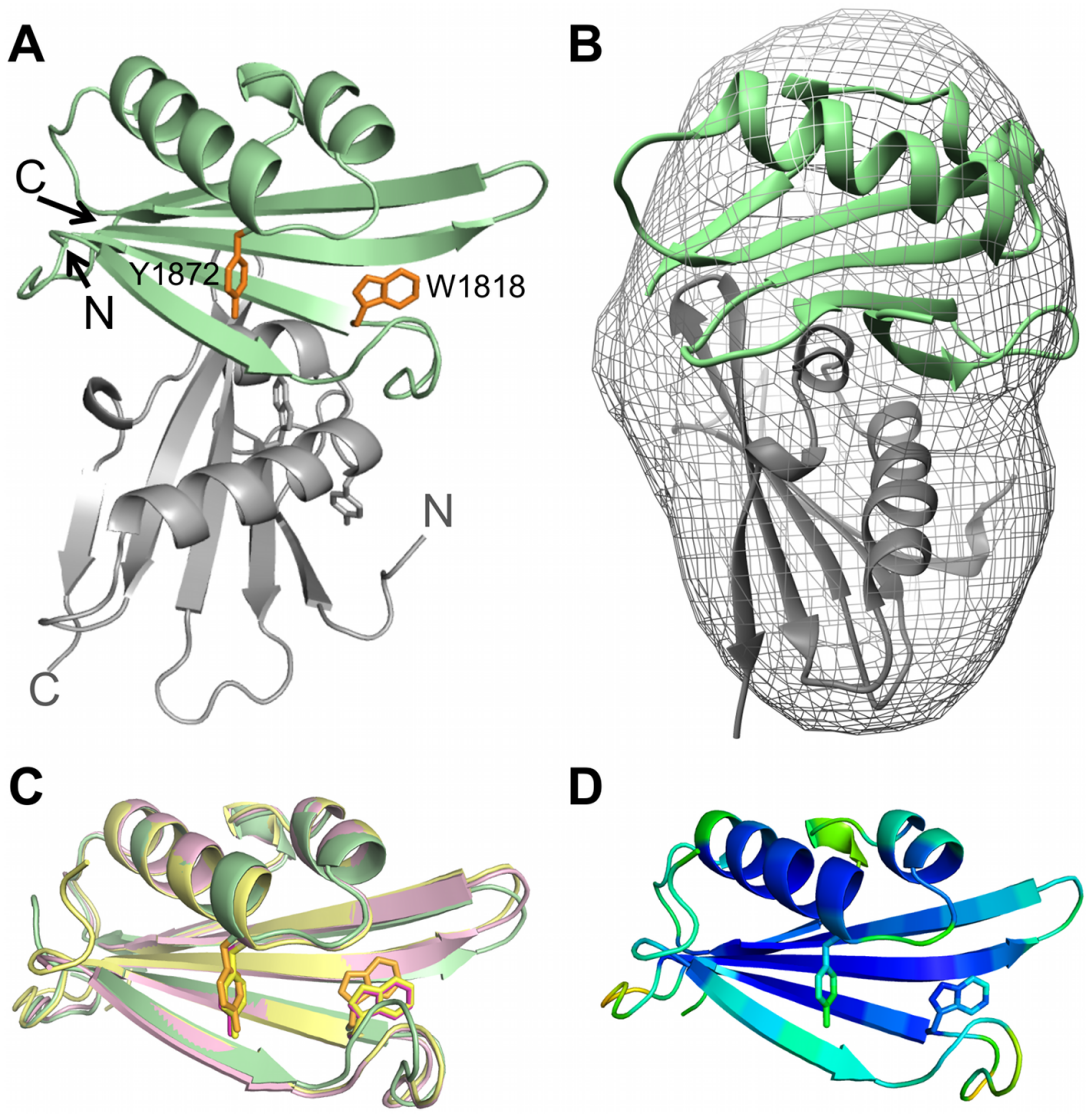
Atomic structure of isolated CASV L-Cterm domain 2. **A)** Ribbon diagram of CASV L-Cterm domain 2 structure. Chain A is shown in palegreen, chain B in grey. The N- and C-termini are marked and potential cap-binding aromatic sidechains Y1872 and W1818 are shown as sticks and colored in orange. **B)** Superimposition of SAXS derived molecular shape of L-Cterm at a concentration of 4.5 mg/ml and ribbon diagram of crystal structure. **C)** Superimposition of ribbon diagrams of chain A and B from isolated CASV L-Cterm domain 2 crystal structure (magenta and yellow, respectively) and L-Cterm crystal structure (green). Potential cap-binding aromatic sidechains are highlighted with saturate colors. **D)** Representation of chain B of L-Cterm domain 2 colored by B-factor with the highest observed B being 106 (orange) and the lowest 22 (dark blue).

To further substantiate that L-Cterm domain 1 has no influence on the conformation of L-Cterm domain 2, we crystallized and solved the structure of the isolated domain 2 (Fig 5A, S1 Table). This structure was refined to a resolution of 1.8 Å. CASV L-Cterm domain 2 also crystallized as a dimer but—due to absence of domain 1—with a completely different and much smaller interface compared to CASV L-Cterm. The protein also appeared as a dimer in solution as shown by SAXS (Fig 5B and S1B Fig). Superimposition of the isolated Cterm domain 2 with its counterpart in the full CASV L-Cterm structure shows only small differences in the loop upstream of W1818 and no major rearrangement of potential cap-binding side chains, even though B-factors are relatively high around the putative cap-binding site (Fig 5C and 5D). Co-crystallization of the domain with m^7^GpppG, m^7^GTP, GTP or ATP did not result in additional electron density.

Again, we did not detect binding to m^7^GTP-agarose of the isolated CASV L-Cterm domain 2 (S6A Fig) nor a thermal stabilization of the protein by m^7^GTP (S7 Fig). Assuming that the cap-structure alone might not be sufficient for binding, we also carried out binding experiments in a native gel using capped RNA. We detected a shift of the RNA with PB2, but not with L-Cterm domain 2 (S6B Fig).

As neither a monomeric form of CASV L-Cterm (deltaC) nor a dimeric form with a different dimer interface (domain 2) binds m^7^GTP, we conclude that the dimerization of the protein and the presence of domain 1 are not responsible for the lack of cap-binding activity.

### Determination of the CASV endonuclease structure

The cap-snatching mechanism has been proposed and characterized so far only for mammarenaviruses based on (i) sequencing results showing 4–5 non-templated nucleotides at the 5' end of viral mRNAs and (ii) structural and functional data demonstrating the existence of an endonuclease in the N-terminus of the L protein [4, 5, 16]. Therefore, we aimed to provide additional evidence for a cap-snatching machinery in reptarenaviruses. We focused on the N-terminus of the L protein, where the endonuclease should be located.

**Fig 6:**
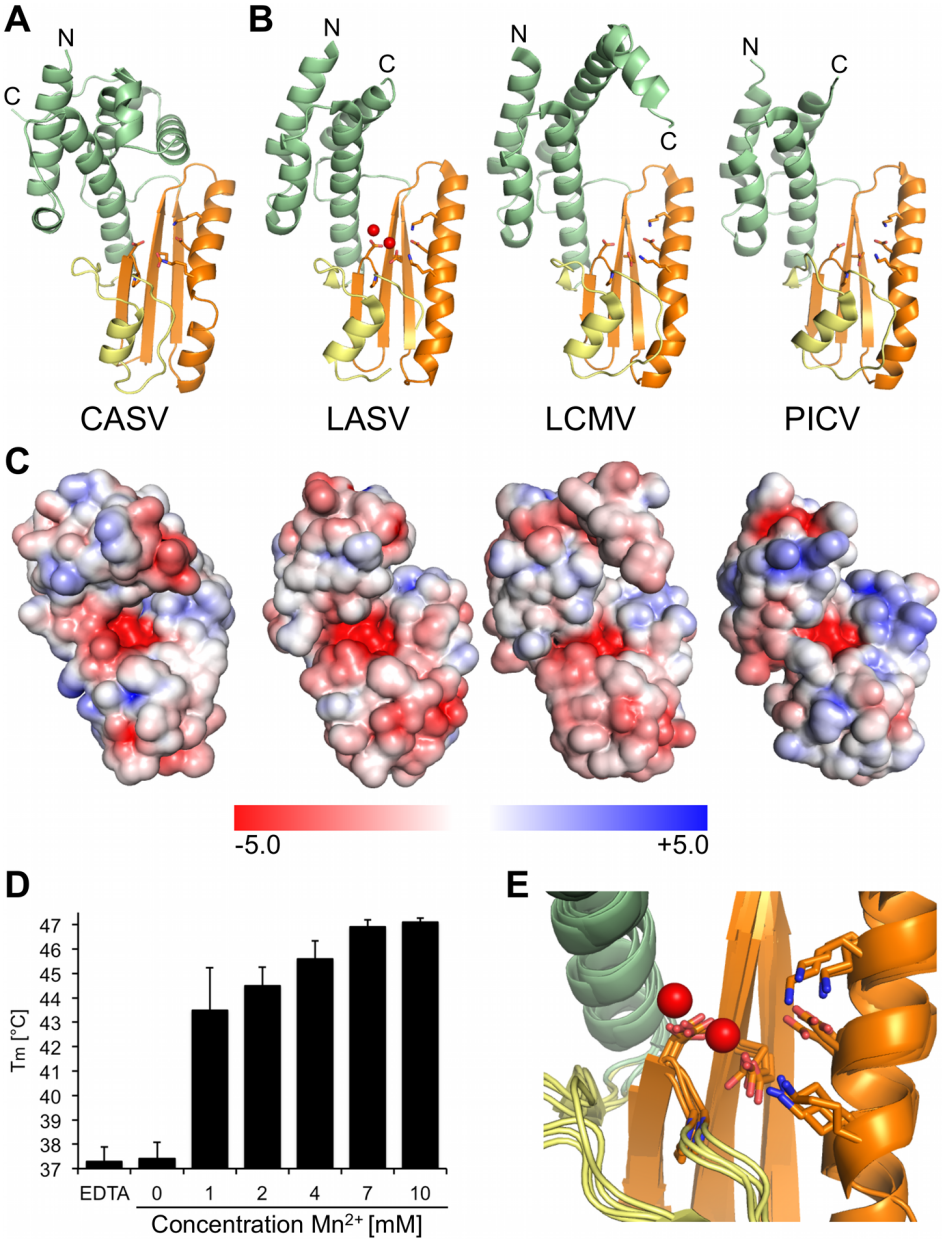
Structure and thermal stabilization of CASV endonuclease. **A)** Ribbon diagram of the CASV endonuclease crystal structure. N- and C-termini are labelled and active site residues are shown as sticks. The conserved β-sheet and the long α-helix are colored in orange, a conserved helix-bundle domain in green and the remaining part with loops and α-helices in yellow. **B)** Endonuclease structures of LASV (PDB ID 5J1P), LCMV (PDB ID 3JSB) and PICV (PDB ID 4I1T) are shown as ribbon diagrams and colored according to CASV endonuclease in A). Manganese ions of LASV structure 5J1P are shown as red spheres, active site residues are shown as sticks, and N- and C-termini are marked. **C)** Electrostatic surface potential of the endonuclease structures shown in A) and B). The surface potential is shown from -5 KT/e in red to +5 KT/e in blue and was calculated using PDB2PQR and the APBS-tool of PyMOL. **D)** Thermal stability of CASV endonuclease depending on Mn^2+^ concentration. Melting temperatures are presented as mean and standard deviations of three independent measurements. Stability of the protein was tested in presence of different concentrations of Mn^2+^ and in presence of 10 mM EDTA. **E)** Close-up of the superimposed endonuclease active sites of the structures shown in A) and B). Conserved active site residues are shown as sticks, and manganese ions of LASV structure 5J1P are shown as red spheres.

In a sequence alignment of arenavirus L protein N-termini, the key active site residues of the endonuclease were found to be highly conserved across the virus family, even in reptarenaviruses (S8 Fig). Therefore, we expressed and purified the first 205 residues of the CASV L protein as N-terminally His-tagged protein. As expected from the metal-dependent enzymatic mechanism of viral endonucleases, thermal stability assays showed a concentration dependent stabilization of the protein by manganese ions with an increase in melting temperature of up to ~10°C at a concentration of 10 mM manganese (protein concentration in the assay 4.2 μM) (Fig 6D). After His-tag cleavage, the protein was crystallized and the crystals diffracted to a resolution of 1.9 Å. Molecular replacement using any of the three known arenavirus endonuclease structures or their subdomains as search models was not successful. Therefore we expressed the protein with seleno-methionines and crystallized it after His-tag cleavage in the presence of manganese ions. Phases were determined using the single anomalous dispersion method and used to solve the structure with the dataset from the better diffracting native crystals. The structure was refined to a resolution of 1.9 Å. The native protein crystallized in space group P2_1_2_1_2_1_ with four molecules per asymmetric unit. The structures of the four molecules are very similar with the only difference in the C-terminal 15 residues, which are not visible in all molecules (RMSD between 0.227 and 0.317 Å). The CASV endonuclease has basically the same fold as endonucleases from LASV, Pichinde virus (PICV), and lymphocytic choriomeningitis virus (LCMV) (Fig 6A and 6B, S1 Table) even though the amino acid sequence of this protein is hardly conserved among these viruses (identity ranging between 20 and 55% and similarity ranging between 54 and 79%, S11 Fig). Slight differences between the structures were observed in the long α-helix parallel to the β-sheet (Fig 6A and 6B, α-helix shown in orange), which is separated into two helices in CASV endonuclease domain compared to the other structures, as well as in the helical region shown in green, which is composed of four to six helices of different length and orientation. RMSD between the structures is in the range of 1.372 Å (CASV vs. LCMV) to 1.856 Å (CASV vs. LASV). The highly conserved residues of the endonuclease active site are positioned as in other arenavirus endonuclease structures (Fig 6E). The electrostatic surface potential of CASV endonuclease is also comparable to the other endonuclease structures with positively charged patches next to the negatively charged active site cavity (Fig 6C). We also tested for endonuclease activity using our previously established RNA cleavage assay [17], however, we did not observe enzymatic activity of the isolated domain (S9 Fig).

## Discussion

Cap-snatching was first discovered in influenza virus [8]. The structures of the individual domains responsible, namely the endonuclease in PA and the cap-binding domain in PB2, have been solved [9–11]. From the structure of the complete influenza polymerase a mechanism for cap-snatching and cap-dependent transcription has been proposed [18]. The cap-snatching mechanism is an attractive drug target, because the corresponding functional domains of the polymerase are both essential and virus specific. After the identification of non-templated host-derived sequences at the 5' ends of mRNAs of other segmented negative strand RNA viruses cap-snatching was proposed to be a common mechanism in these viruses [4, 6, 7, 19–24]. However, in contrast to the endonuclease, which has recently been shown to be located at the very N-terminus of the L protein of mammarena-, orthobunya-, and hantaviruses using structural and molecular biological techniques [5, 16, 17, 25, 26], the cap-binding domain has not been identified in any arena- or bunyavirus so far.

We solved the structure of the 326 C-terminal residues of a reptarenavirus L protein. Despite the lack of any significant sequence homology, the domains of this 37-kDa fragment are structurally similar to the cap-binding and adjacent domains of influenza virus PB2 [15]. Both proteins share a common architecture with respect to the linear arrangement of the domains and of the secondary structure elements. The highest degree of similarity is observed between the PB2 cap-binding domain and domain 2 of L-Cterm. Comparison of these two domains led us to identify a potential cap-binding site in L-Cterm. However, this site does not feature the typical sandwich arrangement of two aromatic residues [27]. While one aromatic residue (Y1872) is in a similar position as its putative homologue in PB2, the hairpin, which provides the second aromatic residue in PB2, is missing in CASV. Several attempts to biochemically or structurally verify the presence of a functional cap-binding site failed. In addition, we solved the crystal structure of the corresponding endonuclease in the N-terminus of the reptarenavirus L protein. It shows a typical endonuclease fold as found in other segmented negative strand RNA viruses and an active site topology that is essentially identical to that of known mammarenavirus endonuclease structures [5, 10, 17, 26, 28].

The main question arising from these data is whether the L protein of CASV—and by inference the L protein of other arenaviruses—contains a functional cap-snatching machinery as described for influenza virus polymerase? There is clear evidence from experiments with replicon systems for LASV and LCMV that the endonuclease at the N-terminus of the L protein is essential for virus transcription [5, 25]. The structures obtained for LASV and LCMV endonuclease domains, specifically the conformation of the active sites, indicate the existence of a functional enzyme, even though catalytic activity of the isolated domains is absent or poor compared to the endonucleases of influenza virus or bunyaviruses [5, 10, 17, 26]. The conserved active site topology in the CASV endonuclease structure and the stabilization of the protein by Mn^2+^ are strong arguments for the presence of a functional endonuclease in the L protein of reptarenaviruses, even though, identical to the isolated endonuclease domain of LASV, nuclease activity was undetectable biochemically [26]. As shown for the influenza virus endonuclease, an activation of the enzyme in the context of the complete L protein is conceivable, partly due to enhanced RNA binding [15]. Unfortunately, we cannot provide functional data for the involvement of the CASV endonuclease in viral transcription, as replicon systems for reptarenaviruses are not available. Nevertheless, in conjunction with available evidence from mammarenaviruses [5, 16, 25, 26] we consider the structural data provided here sufficient to claim the existence of a cap-snatching endonuclease in reptarenaviruses, even without biochemical proof.

In contrast to the endonuclease, both structural and biochemical data suggest that the putative cap-binding site in the C-terminus of CASV L protein is not functional. The data obtained with a dimerization deficient mutant and the isolated domain 2 of L-Cterm exclude that the interaction between domains 1 and 2 at the dimerization interface accounts for the absence of a functional cap-binding site.

We could also neither demonstrate binding of C-terminal L protein fragments of mammarenaviruses to m^7^GTP or capped RNA nor the thermal stabilization of these proteins by m^7^GTP (shown for a soluble LASV L-Cterm fragment in S6 and S7 Figs) indicating that the inability to bind cap-structures is not specific for CASV.

In a previous study, we have identified several amino acid residues in the C-terminus of LASV L protein that are critical for viral transcription but dispensable for genome replication [13]. However, the presence of a cap-binding site could not be inferred, as no motif exists to facilitate its identification at sequence level [27]. To correlate this functional data from LASV with our atomic structure of CASV L-Cterm, we attempted to align the primary sequences of both proteins. Unfortunately, this was not feasible due to the extremely low sequence conservation in the C-terminus of arenavirus L proteins (S12 Fig). Therefore, we used predicted secondary structures of LASV and other arenavirus L protein C-termini [29–31] together with the determined secondary structure from the influenza virus PB2 and CASV L-Cterm crystal structures as a guidance to propose a sequence alignment of these viruses (S2 Fig). Although this alignment has to be interpreted with caution, it facilitated inference of LASV counterparts to CASV L protein residues potentially involved in cap-binding and vice versa (S3 Fig). Specifically, residue F2042 in LASV L protein appeared to be the best homolog candidate to Y1872 in CASV L protein and F404 in influenza virus PB2. We tested various LASV L protein mutants with exchanges at this and adjacent positions in the LASV minireplicon system (S3 and S4 Figs, S2 Table). Most importantly, F2042 in LASV L protein could be replaced by the polar and hydrophilic serine without any effect on the transcriptional activity of the L protein. This phenotype is not compatible with a function of this residue in an aromatic sandwich for cap-binding. In addition, several New World arenaviruses lack an aromatic residue in the region corresponding to F2042 in LASV L [13]. On the other hand, the selective defect in transcription observed with LASV L protein mutants W1915E, E2041L, E2041K, and F2042D (S4 Fig) supports our previous findings that the C-terminus of arenavirus L protein is somehow involved in viral transcription [13]. According to the sequence alignment in S3 Fig, residues implicated in LASV transcription map to various regions of both domains 1 and 2 of CASV L-Cterm (S5 Fig). A possible explanation for the transcription defective phenotype of respective mutants is that these residues play a role in the structural integrity of the C-terminus or in interactions with other viral or cellular factors involved in viral transcription. In summary, the CASV L-Cterm structure, the LASV minireplicon data as well as the cap-binding and thermal shift assays collectively point to the absence of a functional cap-binding site in this region.

The clear structural similarities between influenza virus PB2 and CASV L-Cterm are consistent with the phylogenetic relatedness of influenza virus and arenaviruses. The cap-binding function might have been lost during arenavirus evolution, while the domain might have gained or maintained other functions in virus transcription [13]. A similar situation was proposed for Thogoto virus, an insect transmitted orthomyxovirus. Thogoto virus polymerase PA and PB2 subunits contain domains structurally similar to the endonuclease and cap-binding domains of influenza virus polymerase but with amino acid substitutions in both active sites that render them functionally inactive [32]. The hypothesis of a non-functional cap-binding site in CASV would imply that the cap-snatching mechanism of reptarenaviruses, and perhaps arenaviruses in general, is divergent from that of influenza virus. There are indeed significant differences in the transcription initiation between both virus families. Influenza virus depends on nuclear RNA polymerase II as provider of capped host cell RNA [33]. As arenaviruses replicate in the cytoplasm, they must have acquired a different source of cellular capped RNAs. This could involve cellular cap-binding proteins [34], which may substitute for a cap-binding domain in the L protein. Additionally, more than 50% of the arenavirus L protein has neither been structurally characterized nor assigned a distinct function. Thus it is still possible that a different cap-binding site could be present even in the L protein, although in the corresponding region of bunyavirus L protein, no cap-binding domain is apparent [28]. Arenavirus NP has also been proposed as a cap-binding protein [35] although this hypothesis could not be confirmed using the LASV minireplicon system [36] and in the crystal structure of the NP-RNA complex the suggested cap-binding site was shown to be an RNA binding site [37].

An alternative and speculative hypothesis is that the potential cap-binding site in CASV might be able to adopt alternative configurations; the binding site may switch between active and inactive conformations. These may, for example, correspond to transcription and replication mode of the L protein, respectively. The putative cap-binding site in CASV L-Cterm, inactive in isolation, might become activated in the physiological RNP context as a result of interactions with other parts of the L protein, other viral proteins such as NP or Z [38–40], cellular factors, virus RNA and/or host cell RNA. A hypothetical viral or cellular partner could induce a conformational change, which facilitates the formation of a functional cap-binding site. Binding of viral RNA also has a considerable effect on the configuration of the cap-binding and endonuclease domains in the context of the complete influenza virus polymerase complex [15, 41]. Moreover, induced fit is not unknown in cap-binding proteins: for example, the cap-binding side chains of eIF4E undergo significant rearrangement upon ligand binding [42].

In conclusion, we solved the structures of the isolated N- and C-termini of CASV L protein. The N-terminus harbors a presumably active cap-snatching endonuclease, which is structurally similar to its homologs from mammarenaviruses. The C-terminus shows structural similarity to the influenza virus cap-binding protein PB2, although the cap-binding site is not functional in the isolated domain. Our data provide insight into possible scenarios of transcription initiation in arenaviruses. Future experiments in the context of the full-length L protein may elucidate the detailed mechanisms.

## Methods

### Cloning, expression and purification of arenavirus L protein C-terminus

Based on an alignment of arenavirus L protein C-terminal sequences, we designed L protein expression constructs of different lengths for 20 arenavirus species covering the full phylogenetic spectrum. All sequences were cloned into pOPINF vectors [43] using the In-Fusion HD EcoDry Cloning Kit (Clontech). Solubility of fragments was assessed in a medium-throughput setup with different *E*. *coli* strains, autoinduction medium and small-scale His-tag purification and the expression and purification subsequently optimized for soluble proteins. The CASV L-Cterm and domain 2 were expressed in *E*. *coli* strain BL21 Gold (DE3) (Novagen) at 17°C overnight using TB medium and 0.5 mM isopropyl-β-D-thiogalactopyranosid for induction. After pelleting, the cells were resuspended in 50 mM Tris, pH 8.0, 300 mM NaCl, 10 mM imidazole, 0.5 mM phenylmethylsulfonyl fluorid, 0.4% (v/v) triton X-100 and 0.025% (w/v) lysozyme and subsequently disrupted by sonication. The protein was purified from the soluble fraction after centrifugation by Ni affinity chromatography. A buffer containing 50 mM imidazole was used for the washing steps and another buffer with 500 mM imidazole for the elution of the protein. Affinity chromatography was followed by size exclusion chromatography (Superdex 200, 50 mM Tris, pH 7.5, 150 mM NaCl, 10% glycerol, 2 mM dithiothreitol) and removal of the N-terminal His-tag by a GST-tagged 3C protease at 4°C overnight. Furthermore, the protein was purified by anion exchange chromatography (loading buffer: 50 mM Tris, pH 7.5, 100 mM NaCl, elution with salt gradient up to 1M NaCl) and a second size exclusion chromatography (see above). Purified proteins were concentrated using centrifugal devices, flash frozen in liquid nitrogen, and stored in aliquots at –80°C.

### Cloning, expression and purification of CASV endonuclease

Based on an alignment of arenavirus L protein N-terminal sequences, we designed L protein constructs of different lengths for CASV endonuclease. Cloning procedures, solubility testing, and large-scale expression was essentially done as described for CASV L-Cterm constructs. After pelleting, the cells were resuspended in 50 mM Na-phosphate, pH 6.8, 300 mM NaCl, 10 mM imidazole, and Complete protease inhibitor EDTA-free (Roche). *E*. *coli* were disrupted by sonication and the protein was purified by Ni affinity chromatography from the soluble fraction after centrifugation. A buffer containing 50 mM imidazole was used for the washing steps and the protein was eluted by a buffer containing 100 mM Na-phosphate, pH 6.8, 300 mM NaCl and 250 mM imidazole. The His-tag was removed by incubation with a GST-tagged 3C protease at 4°C overnight with simultaneously dialyzing against 20 mM Tris pH 7.5, 100 mM NaCl, 1mM EDTA and 2.5% glycerol. Furthermore, the protein was purified by anion exchange chromatography (elution with salt gradient up to 1M NaCl) and size exclusion chromatography (Superdex 200, 20 mM Na-phosphate, pH 6.0, 300 mM NaCl, and 5% glycerol). Purified proteins were concentrated using centrifugal devices, flash frozen in liquid nitrogen, and stored in aliquots at –80°C.

### Production of seleno-methionine labelled protein

Protein expression was done in M9 minimal medium [44] supplemented with 1 mM MgSO_4_, 0.4% glucose, 0.0005% thiamine and 200 μM FeSO_4_ at 17°C overnight. Incorporation of seleno-methionine was achieved by metabolic inhibition of methionine biosynthesis in *E*. *coli* prior to addition of seleno-methionine and induction with 1 mM isopropyl-β-D-thiogalactopyranosid. Cells were harvested and the labelled protein was purified as described but in presence of 5 mM β-mercaptoethanol for Ni affinity purification and 10 mM dithiothreitol for the remaining purification steps.

### Crystallization and structure determination of CASV L-Cterm and domain 2

The CASV L-Cterm protein was produced with seleno-methionine labelling. Protein crystals grew at 12 mg/ml protein concentration in 37% Jeffamine ED-2001, 2 mM TCEP and 100 mM HEPES pH 7.1 in a sitting drop vapor diffusion setup at 20°C. L-Cterm domain 2 crystallized in presence of 100 mM Tris, pH 7.9, 1.3 M trisodium citrate at 10 mg/ml protein concentration by sitting drop vapor diffusion at 20°C. Crystals were flash frozen in liquid nitrogen with 30% glycerol as cryo protectant. Datasets for CASV L-Cterm were obtained at the ID29 beamline of the ESRF, Grenoble, France. Data for L-Cterm domain 2 crystals were collected at beamlines P13 and P14 of PETRA III at Deutsches Elektronen Synchrotron (DESY), Hamburg, Germany. Datasets were processed with iMosflm [45]. Phases for the CASV L-Cterm structure were determined using the single anomalous dispersion method and PHENIX AutoSol [46] and then used to solve the structure with a new dataset from better diffracting crystals. The L-Cterm domain 2 structure was solved by molecular replacement with the CASV L-Cterm structure using residues 1794–1894 and PHASER [47]. Both structures were refined by iterative cycles of manual model building in Coot [48] and computational optimization with PHENIX [46]. Visualization of structural data was done using PyMOL (PyMOL Molecular Graphics System, Version 1.7 Schrödinger, LLC.) and UCSF Chimera [49]. Electrostatic surfaces were calculated using PDB2PQR and APBS [50, 51].

### Crystallization and structure determination of CASV endonuclease

The CASV endonuclease protein was produced as a native protein (Endo_native_) and with seleno-methionine labelling (Endo_SeMet_), respectively. Protein crystals of the Endo_native_ protein grew at 10 mg/ml protein concentration in 20% PEG 200, 2.5% PEG 3000, and 100 mM MES, pH 5.7, whereas the Endo_SeMet_ protein crystallized in presence of 2% 2-propanol, 8% PEG 4000, 7 mM MnCl_2_ and 100 mM Na-citrate, pH 5.4, at 8 mg/ml protein concentration. Crystals were obtained in a sitting drop vapor diffusion setup at 6–8°C. Crystals were flash frozen in liquid nitrogen with 30% PEG 400 (Endo_native_) or 20% ethylene glycol (Endo_SeMet_) as cryo protectants. Datasets for both proteins were collected at beamlines P13 and P14 of PETRA III at DESY, Hamburg. Datasets were processed with iMosflm [45] and the Endo_SeMet_ structure was solved by the single anomalous dispersion method using PHENIX AutoSol [46]. The Endo_native_ structure was solved by molecular replacement with the Endo_SeMet_ structure using only chain A and PHASER [47]. Refinements, visualization of structures and calculation of electrostatic surface potentials was done as for CASV L-Cterm.

### Thermal stability assay

The thermal stability of CASV endonuclease was measured by thermofluor assay [52]. The assay contained a final concentration of 4.2 μM of the endonuclease protein, 100 mM Tris, pH 7.5, 150 mM NaCl, SYPRO-Orange (final dilution 1:1000) and either 10 mM EDTA, various concentrations of MnCl_2_ or no further additives.

Thermal stability of CASV L-Cterm proteins, LASV L-Cterm and Influenza virus PB2 was tested in presence and absence of m^7^GTP, GTP and ATP. The final protein concentration in these assays was between 4 and 17 μM (CASV L-Cterm 5.3 μM, L-Cterm deltaC 5.6 μM, L-Cterm domain 2 17.0 μM, LASV L-Cterm 4.1 μM and PB2 10.6 μM). Reactions were carried out in 100 mM Tris, pH 7.5, 150 mM NaCl and SYPRO-Orange.

### Cap-binding pull-down assay

Proteins were incubated overnight at 4°C or for 2 h at 20°C at a concentration of 50 μg/ml with m^7^GTP-agarose or blank agarose (both Jena Bioscience), respectively, in a buffer containing 50 mM Tris, pH 7.5, 150 mM NaCl, 10% glycerol, and 0.005% Tween 20. Agarose beads were washed extensively with the mentioned buffer and SDS sample buffer was added to the beads for subsequent SDS-PAGE analysis.

### Radioactive electrophoretic mobility shift assay

A 40mer polyA RNA substrate was produced by *in vitro* transcription and radioactively labelled by capping with capping enzymes (Cellscript) and α^32^P-GTP. In parallel a synthetic polyA 40mer RNA was labelled with T4 polynucleotide kinase (New England Biolabs) and γ^32^P-ATP. RNA substrates were subsequently purified with a Microspin G25 column (GE Healthcare). Reactions containing 5 pmol of protein and 0.4 pmol total RNA (fraction of radioactively labelled RNA was constant in all reactions and adjusted to facilitate proper detection) were set up in presence of 0.5 U/μl RNasin (Promega), 20 mM HEPES, pH 7.3, 70 mM KCl, 5 mM MgCl_2_, 0.7 mM dithiothreitol, 15% glycerol and 0.7 μg/μl bovine serum albumin, and incubated for 45 min at 20°C. Samples were subjected to native gel electrophoresis using 4% polyacrylamide Tris-borate-EDTA gels and 0.5-fold Tris-borate buffer. The temperature of the gel during electrophoresis was kept low. Signals were visualized by phosphor screen autoradiography using a Typhoon scanner (GE Healthcare).

### Small angle X-ray scattering

Small angle X-ray scattering (SAXS) measurements were performed after size exclusion chromatography in the respective buffers mentioned in the protein purification procedures with different protein concentrations (typically 0.5–5 mg/ml). Data was collected at the SAXS beamline P12 of PETRA III storage ring of the DESY, Hamburg, Germany [53]. Using a PILATUS 2M pixel detector at 3.1 m sample distance and 10 keV energy (λ = 1.24 Å), a momentum transfer range of 0.01 Å^–1^ < s < 0.45 Å^–1^ was covered (s = 4π sinθ/λ, where 2θ is the scattering angle). Data were analyzed using the ATSAS 2.6 package [54]. The forward scattering I(0) and the radius of gyration Rg were extracted from the Guinier approximation calculated with the AutoRG function within PRIMUS [55]. GNOM [56] provided the pair distribution function P(r) of the particle, the maximum size Dmax and the Porod volume. *Ab initio* reconstructions were generated with the program DAMMIF [57]. Ten independent DAMMIF runs were superimposed by SUPCOMB [58] and averaged using the program DAMAVER [57]. The average excluded volume was extracted from the final pdb-file. Structures were visualized using UCSF Chimera.

## Supporting information

S1 Fig Supplementary data for SAXS experiments. **A)** Comparison of experimental scattering curves (grey dots) and theoretical scattering curves for the CASV L-Cterm structure (red line). χ2-value is given. The theoretical curve was calculated and fit to the experimental data using CRYSOL [59]. **B)** Comparison of experimental scattering curves (grey dots) and theoretical scattering curves for the CASV L-Cterm domain 2 structure (red line). χ2-value is given. The theoretical curve was calculated and fit to the experimental data using CRYSOL. **C)** Plot of experimental scattering data for CASV L-Cterm (black) and L-Cterm deltaC mutant (red) measured at equal concentrations. The table shows the calculated molecular weight (MW) from SAXS data (derived from Porod volume and average excluded volume of the DAMFILT [57] model) in comparison to the actual MW of the proteins.
(TIF)

S2 Fig Alignment of arenavirus C-terminal sequences and influenza virus PB2. The alignment was created by manually combining results of PRALINE, MUSCLE, ClustalOmega and Jpred4 programs [29–31, 60]. It initially included L protein sequences and secondary structure predictions from 46 mammarena- and reptarenaviruses, which were reduced to eight sequences for a better overview. After adding influenza virus PB2 sequence the alignment was further adjusted manually. Finally the alignment includes sequences from L proteins of reptarenaviruses CASV (Uniprot-ID: J7HBG8) and Boa arenavirus NL (ROUTV, M4PUV6) and mammarenaviruses LASV (Q6Y630), Mobala virus (MOBV, Q27YE5), LCMV (P14240), Junin virus (JUNV, Q6XQI4), Tacaribe virus (TACV, P20430) and Oliveros virus (OLVV, Q6XQH7) as well as a sequence of influenza A virus PB2 (FluA, Q6DNN3). The N- and C-termini of CASV L-Cterm domain 2 are marked with red triangles. The potential cap-binding aromatic residues of CASV are marked with an orange asterisk. The conserved C-terminal tail of arenaviruses is highlighted with a yellow box. The secondary structure from the CASV L-Cterm crystal structure (CASV Xtal) is shown above the sequences. Secondary structures as predicted by Jpred4 are shown below the sequences. The secondary structure from influenza virus PB2 crystal structure (FluA Xtal, PDB ID 5FMM) is shown at the bottom. The alignment was drawn using the ESPript online tool (http://espript.ibcp.fr) [61] with manual adjustments.
(TIF)

S3 Fig Residues in LASV L protein functionally tested in the LASV minireplicon system aligned with their putative homologues in other arenaviruses. The alignment is identical to that presented in S2 Fig. Residues in LASV L protein that were mutated and tested in the LASV minireplicon system (S1 Methods) are marked together with their putative homologs in other arenaviruses. Residues identified as important for transcription of LASV in this and a previous study [13] are highlighted in orange, while residues without a specific role during viral transcription are marked in grey.
(TIF)

S4 Fig Minireplicon data for LASV L protein mutants. Transcriptional activity of L protein mutants was measured via Ren-Luc reporter gene expression. The Ren-Luc activity is shown in the bar graph (mean and standard deviation of standardized relative light units [sRLU] as a percentage of the wild-type in ≥3 independent transfection experiments). Synthesis of the antigenome and Ren-Luc mRNA was evaluated by Northern blotting using a radiolabeled riboprobe hybridizing to the Ren-Luc gene. A defective L protein with a mutation in the catalytic site of the RNA-dependent RNA polymerase served as a negative control (neg). Signals on Northern blots were quantified via intensity profiles. The data are also presented numerically in S2 Table. The methylene blue-stained 28S rRNA is shown as a marker for gel loading and RNA transfer. Immunoblot analysis of FLAG-tagged L protein mutants is shown. Mutants with an mRNA defective phenotype are marked with an asterisk. For experimental details see S1 Methods.
(TIF)

S5 Fig Linking the LASV replicon data with the CASV L-Cterm structure. Ribbon diagram of CASV L-Cterm structure in **A)** front sight and **B)** back sight. Residues that were found to be important for viral transcription in LASV minireplicon system in this and a previous study [13] and could be located in the CASV L-Cterm structure according to the alignment in S3 Fig are shown as red sticks. **C)** Summary of residues important for LASV transcription. The table further lists the putative equivalent residues in CASV, their location in either domain 1 or 2 of CASV L-Cterm and proposes a function of these residues within the CASV L-Cterm structure.
(TIF)

S6 Fig Cap-binding assays. **A)** Assay for binding to m^7^GTP-agarose. The figure shows coomassie stained SDS gels including the molecular weight marker (MW). For every protein tested the gel contains three lanes: the protein to be used for the assay (first lane), the fraction bound to m^7^GTP-agarose (second lane, m^7^GTP) and the fraction bound to blank agarose as a specificity control (third lane, control). eIF4E and influenza virus PB2 were used as positive controls for m^7^GTP-agarose binding. CASV L-Cterm (Cterm), L-Cterm domain 2 (domain 2), L-Cterm deltaC (deltaC) and LASV L-Cterm were tested at 4°C and 20°C. **B)** Assay for binding to capped RNA. Radioactively labelled capped RNA was incubated with either influenza virus PB2 (PB2), CASV L-Cterm (Cterm), CASV L-Cterm deltaC (deltaC), CASV L-Cterm domain2 (domain 2), LASV L-Cterm (LASV C) or no protein (neg). Free RNA and protein-RNA complexes were separated in a native gel and visualized by autoradiography. **C)** Assay to test for RNA binding independent of a cap-structure for CASV L-Cterm deltaC. CASV L-Cterm deltaC (deltaC) was incubated with different amounts of capped (cap-RNA) or non-capped RNA (RNA) in presence of radioactively labelled non-capped RNA (^32^P-RNA). Total amounts of RNA were kept constant in all reactions. Free RNA and protein-RNA complexes were separated in a native gel and visualized by autoradiography.
(TIF)

S7 Fig Thermal stability assay in presence of m^7^GTP, GTP or ATP. Thermal stability of the proteins CASV L-Cterm, CASV L-Cterm deltaC, CASV L-Cterm domain 2, influenza virus PB2 and LASV L-Cterm was measured in absence (control) and presence of 2, 5, and 10 mM of either m^7^GTP, GTP or ATP. The presented curves show the relative increase of the fluorescence signal (which is related to protein unfolding) as a function of the temperature. A difference of at least 3°C at 50% fluorescence level (dashed line in grey) indicates a significant change in the thermal stability of the protein.
(TIF)

S8 Fig Alignment of arenavirus N-terminal sequences and influenza PA. The alignment was generated using ClustalOmega [31] with manual adjustments. It includes sequences from L proteins of reptarenaviruses CASV (Uniprot-ID: J7HBG8) and Boa arenavirus NL (ROUTV, M4PUV6) and mammarenaviruses LASV (Q6Y630), Mobala virus (MOBV, Q27YE5), LCMV (P14240), Junin virus (JUNV, Q6XQI4), Tacaribe virus (TACV, P20430) and Oliveros virus (OLVV, Q6XQH7) as well as a sequence of influenza A virus PA (FluA, P31343). The key active site residues of the endonuclease are marked with red triangles. The secondary structure of the CASV endonuclease crystal structure (CASV Xtal) is shown above the sequences. Secondary structures predicted by Jpred4 [60] are shown below the sequences. The secondary structure from influenza virus PA crystal structure (FluA Xtal, PDB ID 2W69) is shown at the bottom. The alignment was drawn using the ESPript online tool (http://espript.ibcp.fr) [61] with manual adjustments.
(TIF)

S9 Fig Endonuclease assay for CASV Endo. The activity of the CASV endonuclease was tested in our previously published radioactive endonuclease assay (S1 Methods)[17]. ^32^P-labeled polyA ssRNA substrates of two different lengths (27 and 40 nucleotides) were incubated for 1 h at 37°C either in presence or absence of 5 mM Mn^2+^ with a catalytically active Andes virus endonuclease mutant (ANDV Endo_K44A_), a catalytically inactive Andes virus endonuclease mutant (ANDV Endo_H36R_) or CASV endonuclease fragment (CASV Endo). Substrates and reaction products were separated in a denaturing polyacrylamide gel and visualized by autoradiography.
(TIF)

S10 Fig Electrostatic surface potential of CASV L-Cterm. Acidic and basic amino acid patches from the C-terminus, which interlock with each other in the protein dimer, are marked with dashed circles. The electrostatic surface potential is shown from -5 KT/e in red to +5 KT/e in blue and was calculated using PDB2PQR and the APBS-tool of PyMOL.
(TIF)

S11 Fig Comparison of N-terminal sequences of L proteins and PA. **A)** Identity matrix and **B)** similarity matrix of N-terminal sequences. Matrices were calculated based on the presented alignment of N-termini (S8 Fig) using the SIAS online tool (http://imed.med.ucm.es/Tools/sias.html) and values are given in percent relative to the mean length of sequences compared. Abbreviations: Full virus names are given in legend to S8 Fig.
(TIF)

S12 Fig Comparison of C-terminal sequences of L proteins and PB2. **A)** Identity matrix and **B)** similarity matrix of C-terminal sequences. Matrices were calculated based on the presented alignment of C-termini (S2 Fig) using the SIAS online tool (http://imed.med.ucm.es/Tools/sias.html) and values are given in percent relative to the mean length of sequences compared. Abbreviations: Full virus names are given in legend to S2 Fig.
(TIF)

S13 Fig Alignment of reptarenavirus N-terminal sequences. The alignment was generated using ClustalOmega [31] and includes sequences from L proteins of reptarenaviruses CASV (Uniprot-ID: J7HBG8), Boa arenavirus NL (ROUTV, M4PUV6) as well as 13 other reptarenavirus L protein sequences (Uniprot-IDs are given). The secondary structure of the CASV endonuclease crystal structure (CASV Xtal) is shown above the sequences. The alignment was drawn using the ESPript online tool (http://espript.ibcp.fr) [61].
(TIF)

S14 Fig Alignment of reptarenavirus C-terminal sequences. The alignment was generated using ClustalOmega [31] and includes sequences from L proteins of reptarenaviruses CASV (Uniprot-ID: J7HBG8), Boa arenavirus NL (ROUTV, M4PUV6) as well as 13 other reptarenavirus L protein sequences (Uniprot-IDs are given). The secondary structure of the CASV L-Cterm crystal structure (CASV Xtal) is shown above the sequences. The alignment was drawn using the ESPript online tool (http://espript.ibcp.fr) [61] with manual adjustments.
(TIF)

S1 Table Crystallographic data and refinement statistics. (DOC)

S2 Table Analysis of L protein mutants in Lassa virus replicon system. (DOC)

S3 Table List of tested L protein C-term fragments. This list contains tested fragments that were either insoluble, not suitable for crystallization trials or could not be crystallized successfully.
(DOC)

S1 Methods Detailed description of LASV minireplicon experiments and the endonuclease assay. The methods of LASV minireplicon system and the endonuclease assay are described in detail.
(DOC)
