# Supplementary figures and images for: Structural insights into reptarenavirus cap-snatching machinery
Source: PLoS Pathog. 2017 May 15;13(5):e1006400. doi: 10.1371/journal.ppat.1006400 (PMC5444859; doi:10.1371/journal.ppat.1006400)

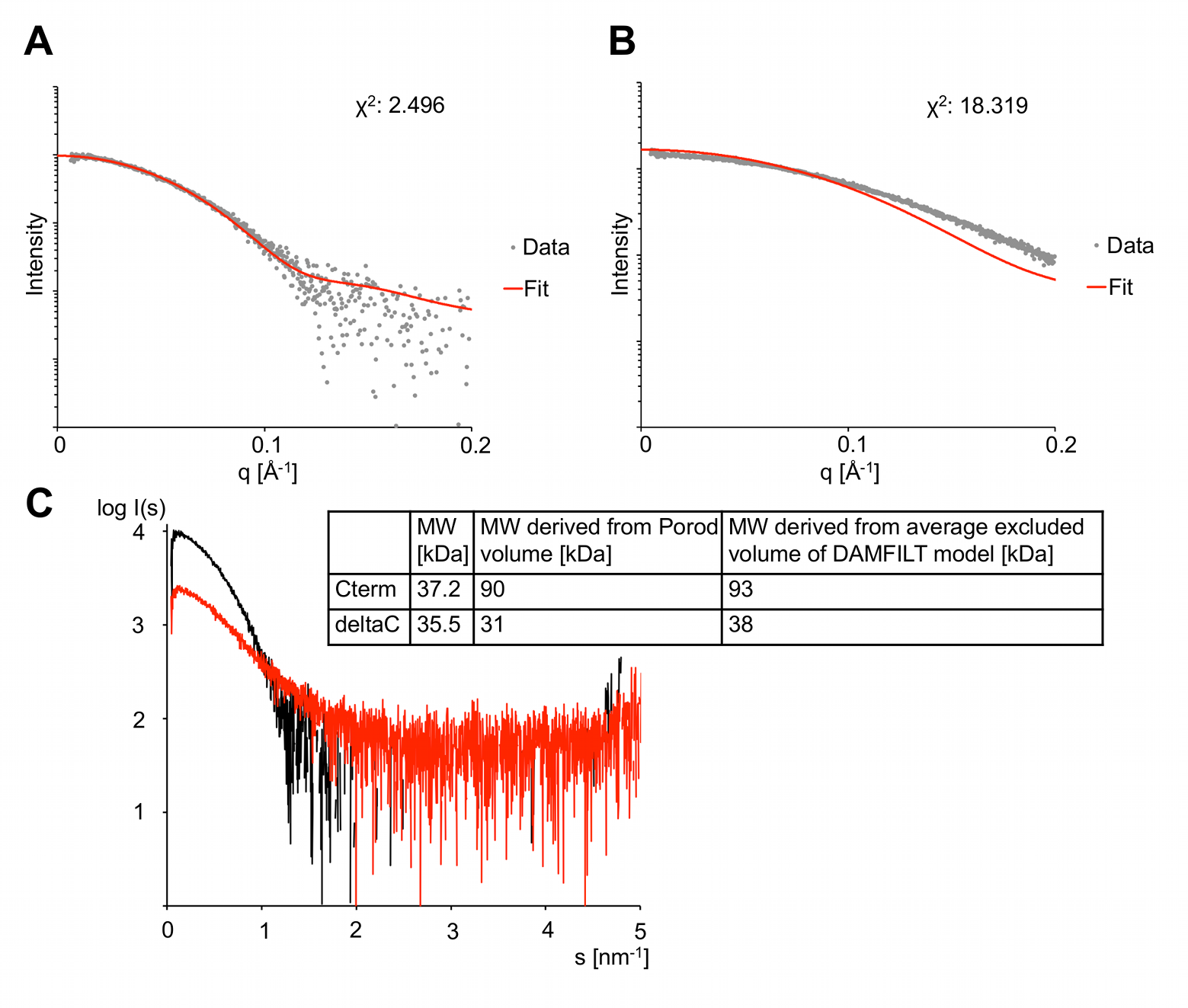

Supplement: S1 Fig — A) Comparison of experimental scattering curves (grey dots) and theoretical scattering curves for the CASV L-Cterm structure (red line). χ2-value is given. The theoretical curve was calculated and fit to the experimental data using CRYSOL [59]. B) Comparison of experimental scattering curves (grey dots) and theoretical scattering curves for the CASV L-Cterm domain 2 structure (red line). χ2-value is given. The theoretical curve was calculated and fit to the experimental data using CRYSOL. C) Plot of experimental scattering data for CASV L-Cterm (black) and L-Cterm deltaC mutant (red) measured at equal concentrations. The table shows the calculated molecular weight (MW) from SAXS data (derived from Porod volume and average excluded volume of the DAMFILT [57] model) in comparison to the actual MW of the proteins. (TIF) [file ppat.1006400.s001.tif]

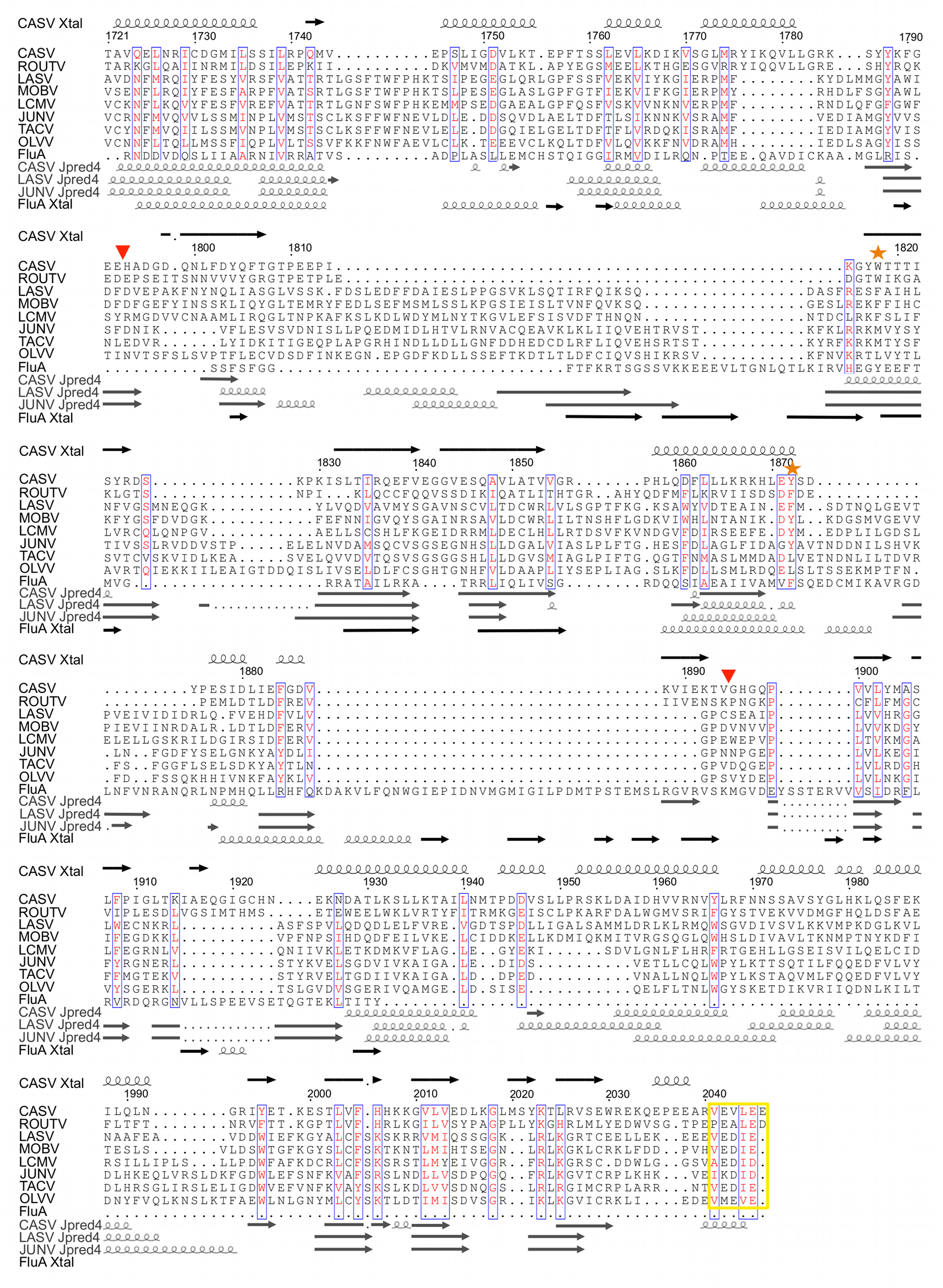

Supplement: S2 Fig — The alignment was created by manually combining results of PRALINE, MUSCLE, ClustalOmega and Jpred4 programs [29–31, 60]. It initially included L protein sequences and secondary structure predictions from 46 mammarena- and reptarenaviruses, which were reduced to eight sequences for a better overview. After adding influenza virus PB2 sequence the alignment was further adjusted manually. Finally the alignment includes sequences from L proteins of reptarenaviruses CASV (Uniprot-ID: J7HBG8) and Boa arenavirus NL (ROUTV, M4PUV6) and mammarenaviruses LASV (Q6Y630), Mobala virus (MOBV, Q27YE5), LCMV (P14240), Junin virus (JUNV, Q6XQI4), Tacaribe virus (TACV, P20430) and Oliveros virus (OLVV, Q6XQH7) as well as a sequence of influenza A virus PB2 (FluA, Q6DNN3). The N- and C-termini of CASV L-Cterm domain 2 are marked with red triangles. The potential cap-binding aromatic residues of CASV are marked with an orange asterisk. The conserved C-terminal tail of arenaviruses is highlighted with a yellow box. The secondary structure from the CASV L-Cterm crystal structure (CASV Xtal) is shown above the sequences. Secondary structures as predicted by Jpred4 are shown below the sequences. The secondary structure from influenza virus PB2 crystal structure (FluA Xtal, PDB ID 5FMM) is shown at the bottom. The alignment was drawn using the ESPript online tool (http://espript.ibcp.fr) [61] with manual adjustments. (TIF) [file ppat.1006400.s002.tif]

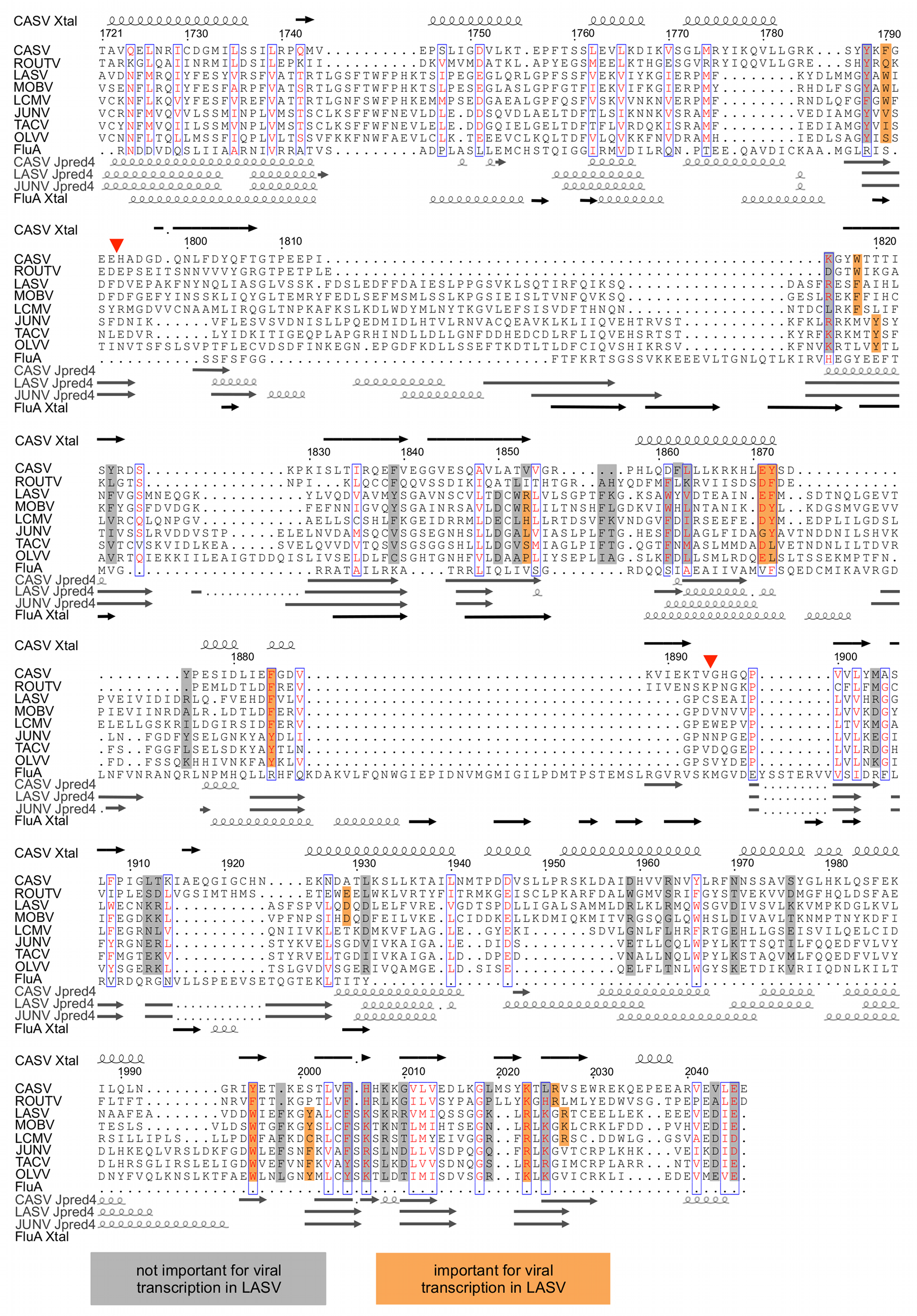

Supplement: S3 Fig — The alignment is identical to that presented in S2 Fig. Residues in LASV L protein that were mutated and tested in the LASV minireplicon system (S1 Methods) are marked together with their putative homologs in other arenaviruses. Residues identified as important for transcription of LASV in this and a previous study [13] are highlighted in orange, while residues without a specific role during viral transcription are marked in grey. (TIF) [file ppat.1006400.s003.tif]

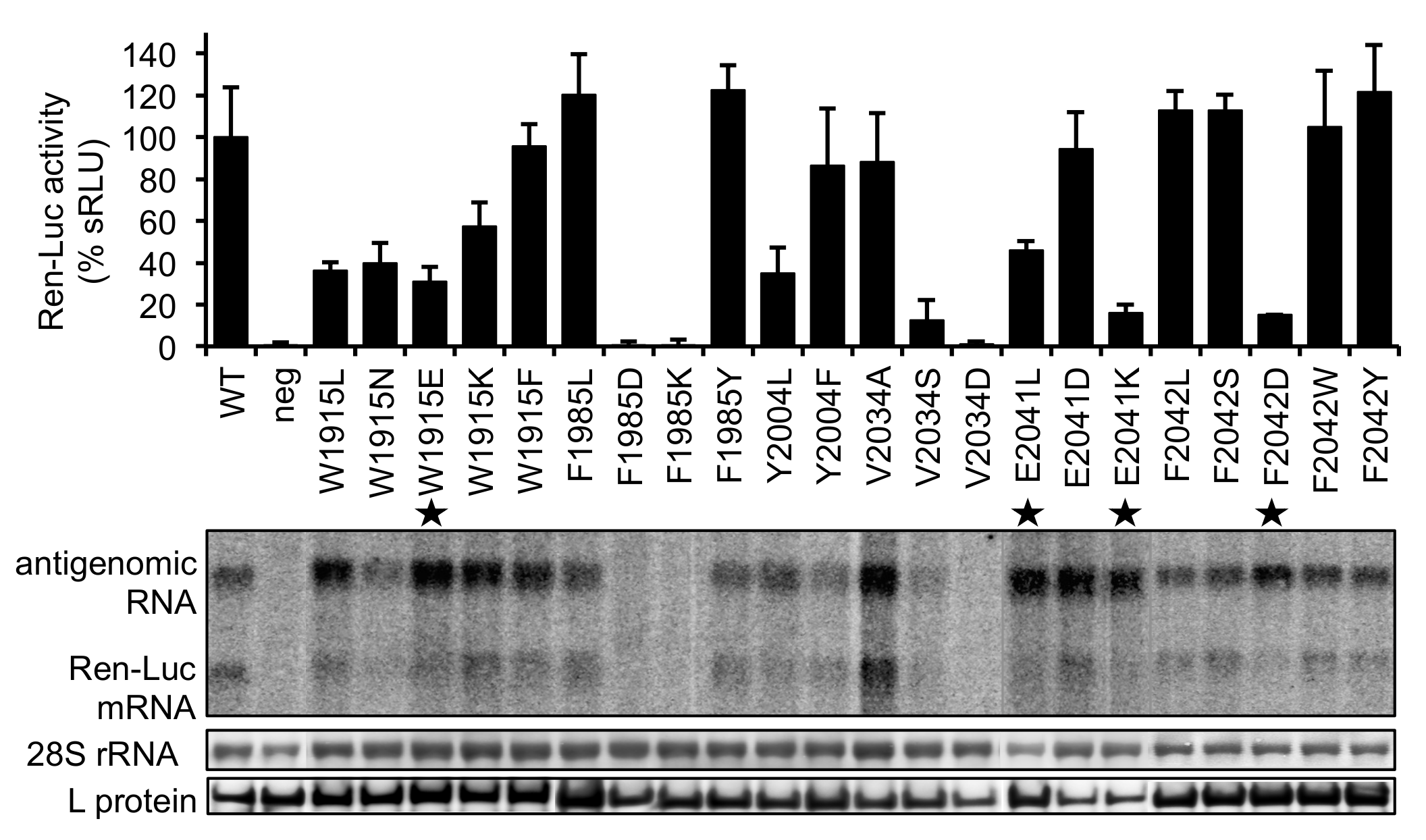

Supplement: S4 Fig — Transcriptional activity of L protein mutants was measured via Ren-Luc reporter gene expression. The Ren-Luc activity is shown in the bar graph (mean and standard deviation of standardized relative light units [sRLU] as a percentage of the wild-type in ≥3 independent transfection experiments). Synthesis of the antigenome and Ren-Luc mRNA was evaluated by Northern blotting using a radiolabeled riboprobe hybridizing to the Ren-Luc gene. A defective L protein with a mutation in the catalytic site of the RNA-dependent RNA polymerase served as a negative control (neg). Signals on Northern blots were quantified via intensity profiles. The data are also presented numerically in S2 Table. The methylene blue-stained 28S rRNA is shown as a marker for gel loading and RNA transfer. Immunoblot analysis of FLAG-tagged L protein mutants is shown. Mutants with an mRNA defective phenotype are marked with an asterisk. For experimental details see S1 Methods. (TIF) [file ppat.1006400.s004.tif]

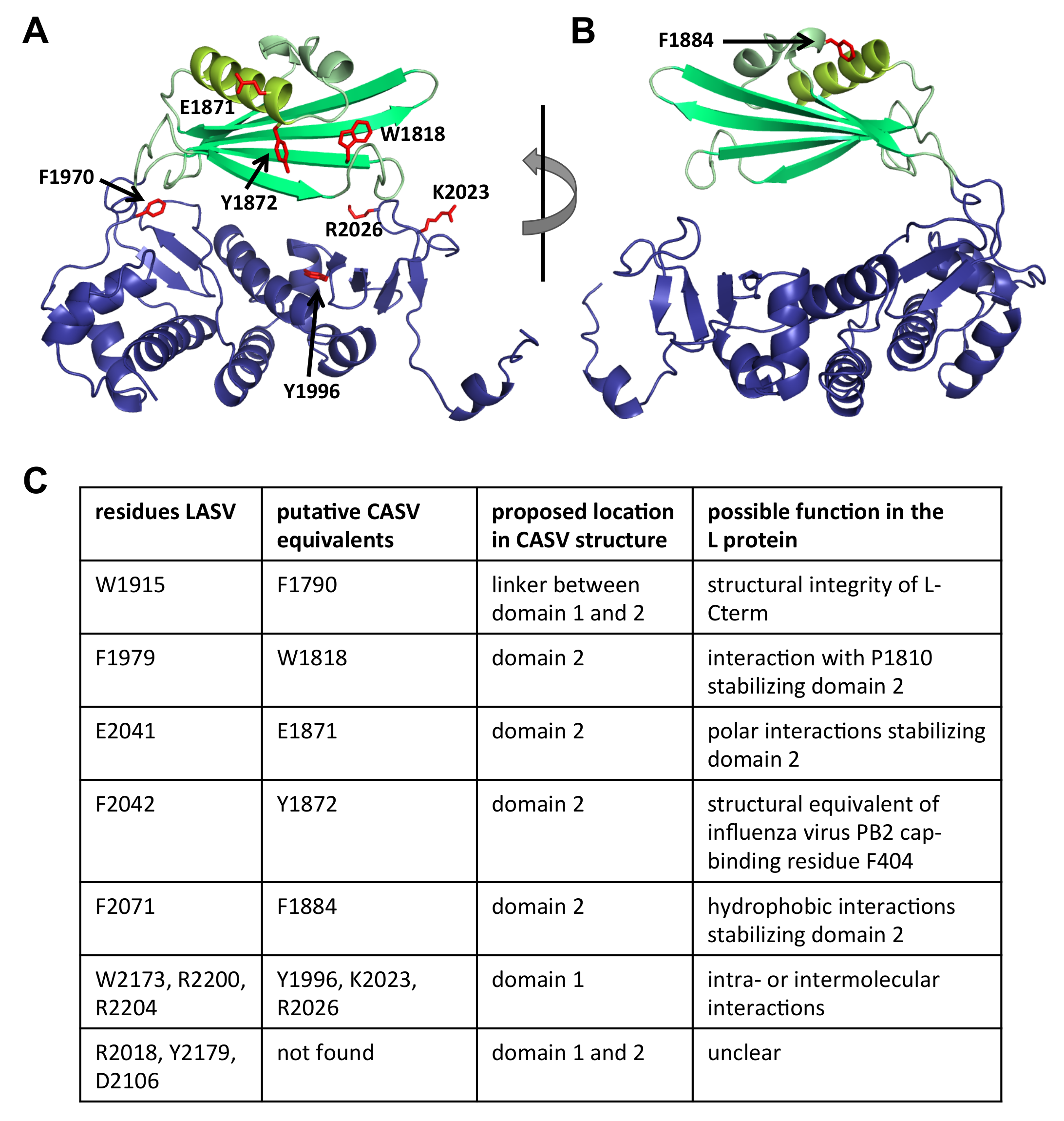

Supplement: S5 Fig — Ribbon diagram of CASV L-Cterm structure in A) front sight and B) back sight. Residues that were found to be important for viral transcription in LASV minireplicon system in this and a previous study [13] and could be located in the CASV L-Cterm structure according to the alignment in S3 Fig are shown as red sticks. C) Summary of residues important for LASV transcription. The table further lists the putative equivalent residues in CASV, their location in either domain 1 or 2 of CASV L-Cterm and proposes a function of these residues within the CASV L-Cterm structure. (TIF) [file ppat.1006400.s005.tif]

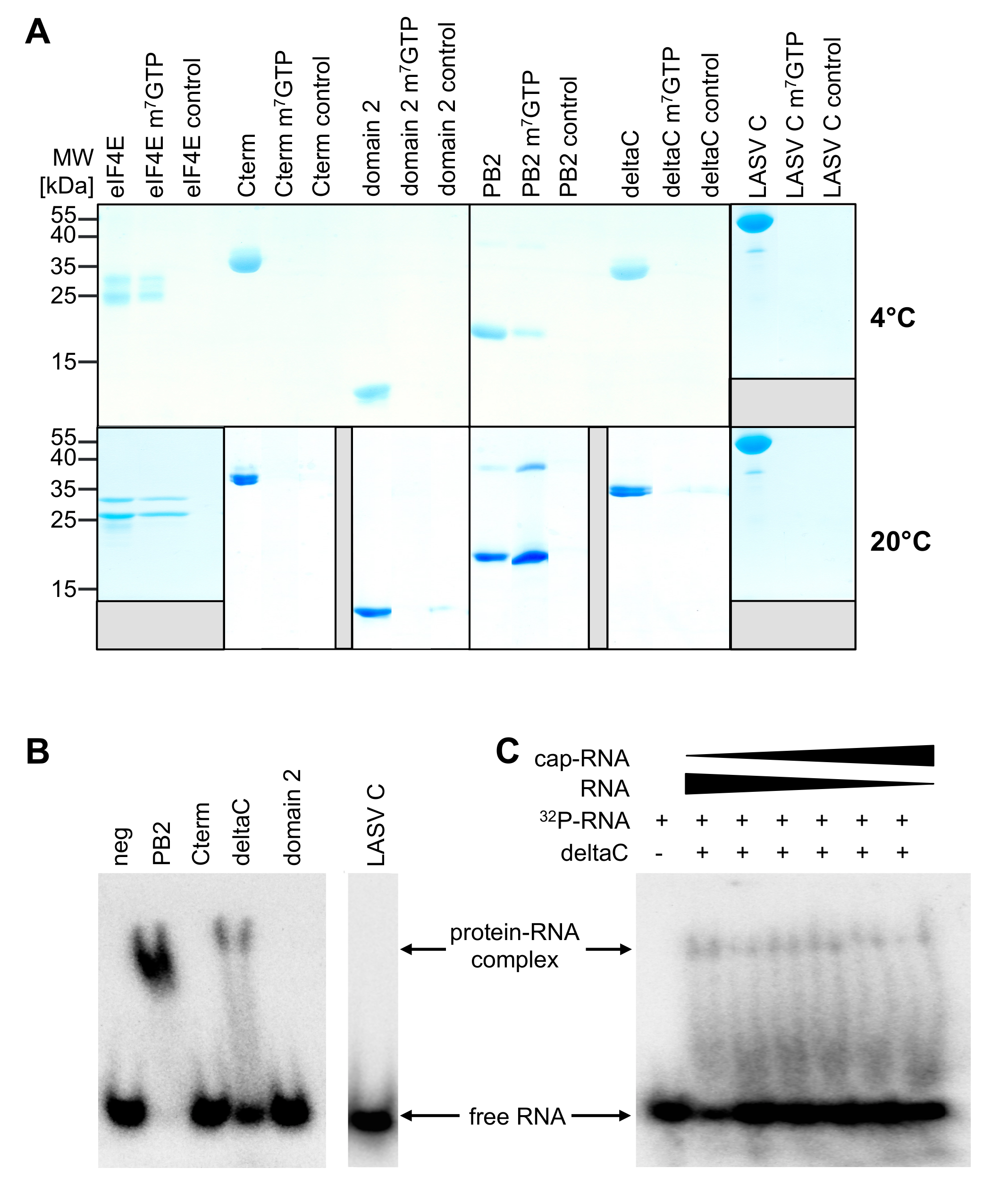

Supplement: S6 Fig — A) Assay for binding to m7GTP-agarose. The figure shows coomassie stained SDS gels including the molecular weight marker (MW). For every protein tested the gel contains three lanes: the protein to be used for the assay (first lane), the fraction bound to m7GTP-agarose (second lane, m7GTP) and the fraction bound to blank agarose as a specificity control (third lane, control). eIF4E and influenza virus PB2 were used as positive controls for m7GTP-agarose binding. CASV L-Cterm (Cterm), L-Cterm domain 2 (domain 2), L-Cterm deltaC (deltaC) and LASV L-Cterm were tested at 4°C and 20°C. B) Assay for binding to capped RNA. Radioactively labelled capped RNA was incubated with either influenza virus PB2 (PB2), CASV L-Cterm (Cterm), CASV L-Cterm deltaC (deltaC), CASV L-Cterm domain2 (domain 2), LASV L-Cterm (LASV C) or no protein (neg). Free RNA and protein-RNA complexes were separated in a native gel and visualized by autoradiography. C) Assay to test for RNA binding independent of a cap-structure for CASV L-Cterm deltaC. CASV L-Cterm deltaC (deltaC) was incubated with different amounts of capped (cap-RNA) or non-capped RNA (RNA) in presence of radioactively labelled non-capped RNA (32P-RNA). Total amounts of RNA were kept constant in all reactions. Free RNA and protein-RNA complexes were separated in a native gel and visualized by autoradiography. (TIF) [file ppat.1006400.s006.tif]

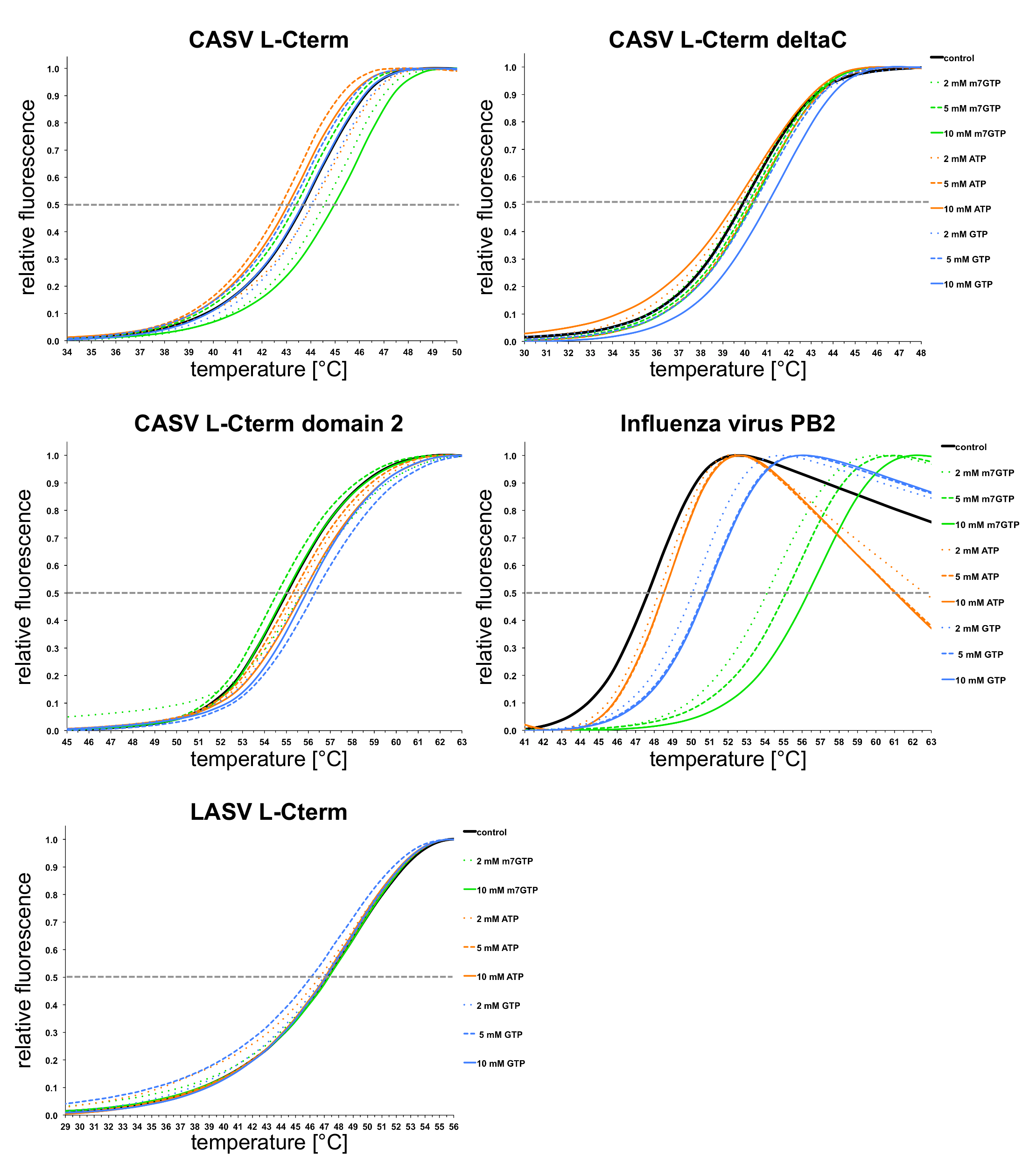

Supplement: S7 Fig — Thermal stability of the proteins CASV L-Cterm, CASV L-Cterm deltaC, CASV L-Cterm domain 2, influenza virus PB2 and LASV L-Cterm was measured in absence (control) and presence of 2, 5, and 10 mM of either m7GTP, GTP or ATP. The presented curves show the relative increase of the fluorescence signal (which is related to protein unfolding) as a function of the temperature. A difference of at least 3°C at 50% fluorescence level (dashed line in grey) indicates a significant change in the thermal stability of the protein. (TIF) [file ppat.1006400.s007.tif]

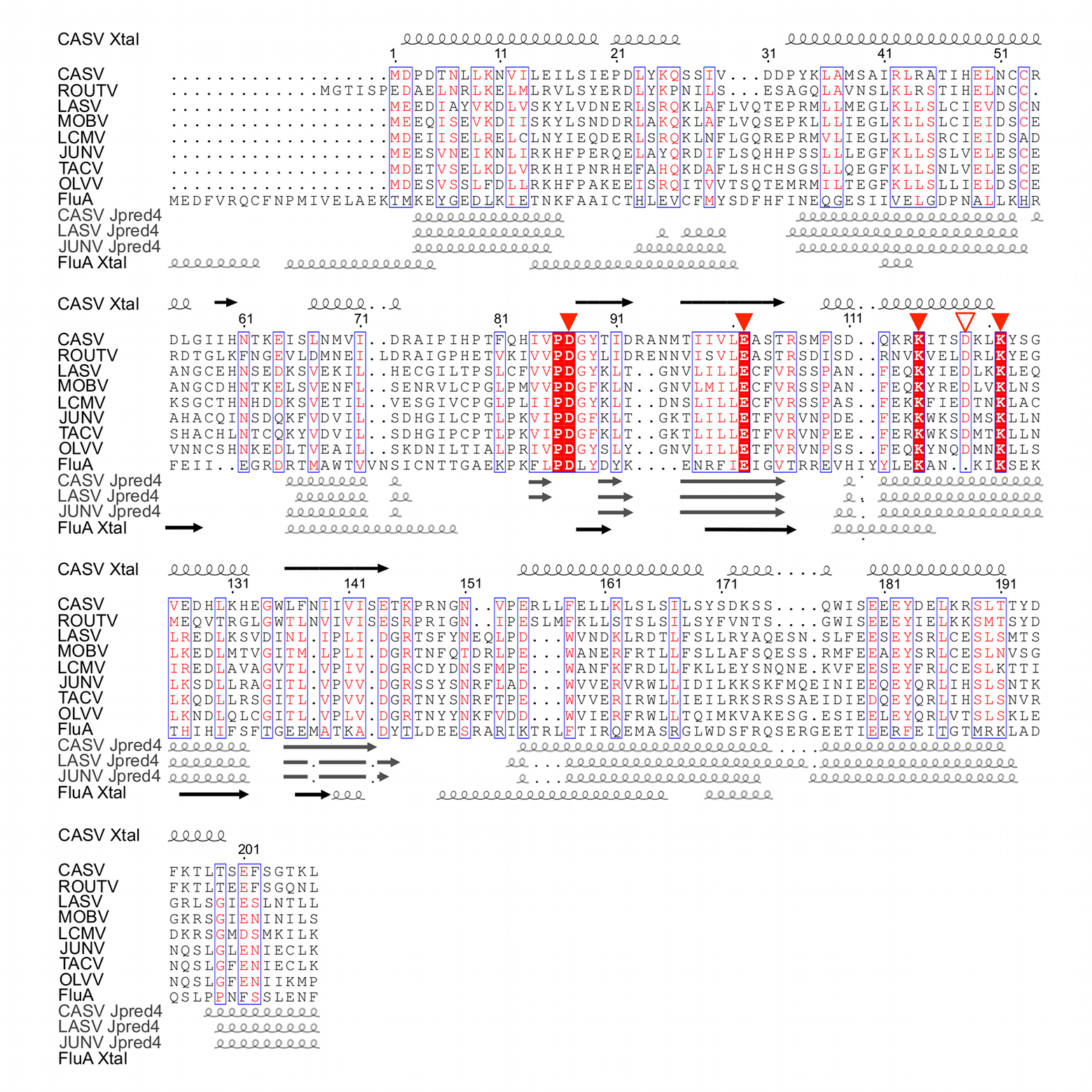

Supplement: S8 Fig — The alignment was generated using ClustalOmega [31] with manual adjustments. It includes sequences from L proteins of reptarenaviruses CASV (Uniprot-ID: J7HBG8) and Boa arenavirus NL (ROUTV, M4PUV6) and mammarenaviruses LASV (Q6Y630), Mobala virus (MOBV, Q27YE5), LCMV (P14240), Junin virus (JUNV, Q6XQI4), Tacaribe virus (TACV, P20430) and Oliveros virus (OLVV, Q6XQH7) as well as a sequence of influenza A virus PA (FluA, P31343). The key active site residues of the endonuclease are marked with red triangles. The secondary structure of the CASV endonuclease crystal structure (CASV Xtal) is shown above the sequences. Secondary structures predicted by Jpred4 [60] are shown below the sequences. The secondary structure from influenza virus PA crystal structure (FluA Xtal, PDB ID 2W69) is shown at the bottom. The alignment was drawn using the ESPript online tool (http://espript.ibcp.fr) [61] with manual adjustments. (TIF) [file ppat.1006400.s008.tif]

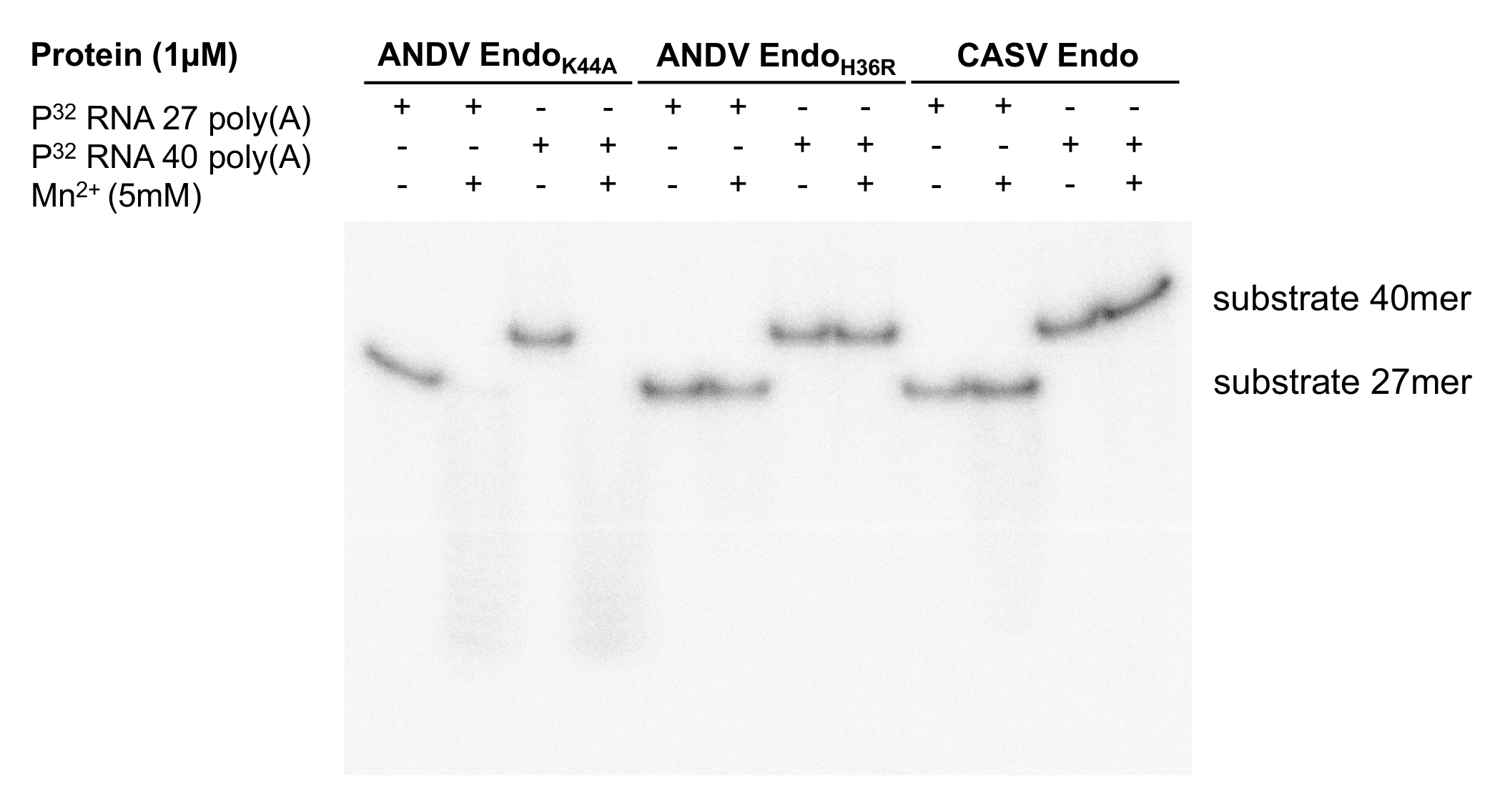

Supplement: S9 Fig — The activity of the CASV endonuclease was tested in our previously published radioactive endonuclease assay (S1 Methods)[17]. 32P-labeled polyA ssRNA substrates of two different lengths (27 and 40 nucleotides) were incubated for 1 h at 37°C either in presence or absence of 5 mM Mn2+ with a catalytically active Andes virus endonuclease mutant (ANDV EndoK44A), a catalytically inactive Andes virus endonuclease mutant (ANDV EndoH36R) or CASV endonuclease fragment (CASV Endo). Substrates and reaction products were separated in a denaturing polyacrylamide gel and visualized by autoradiography. (TIF) [file ppat.1006400.s009.tif]

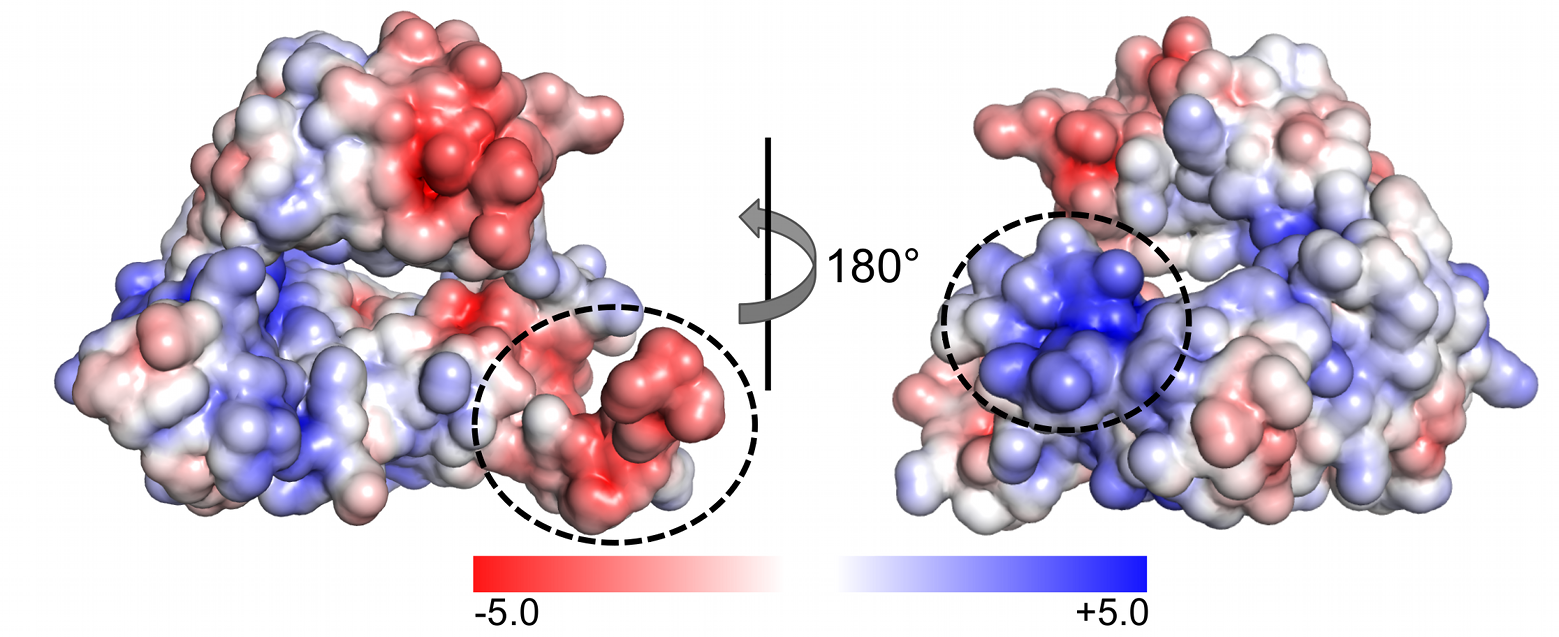

Supplement: S10 Fig — Acidic and basic amino acid patches from the C-terminus, which interlock with each other in the protein dimer, are marked with dashed circles. The electrostatic surface potential is shown from -5 KT/e in red to +5 KT/e in blue and was calculated using PDB2PQR and the APBS-tool of PyMOL. (TIF) [file ppat.1006400.s010.tif]

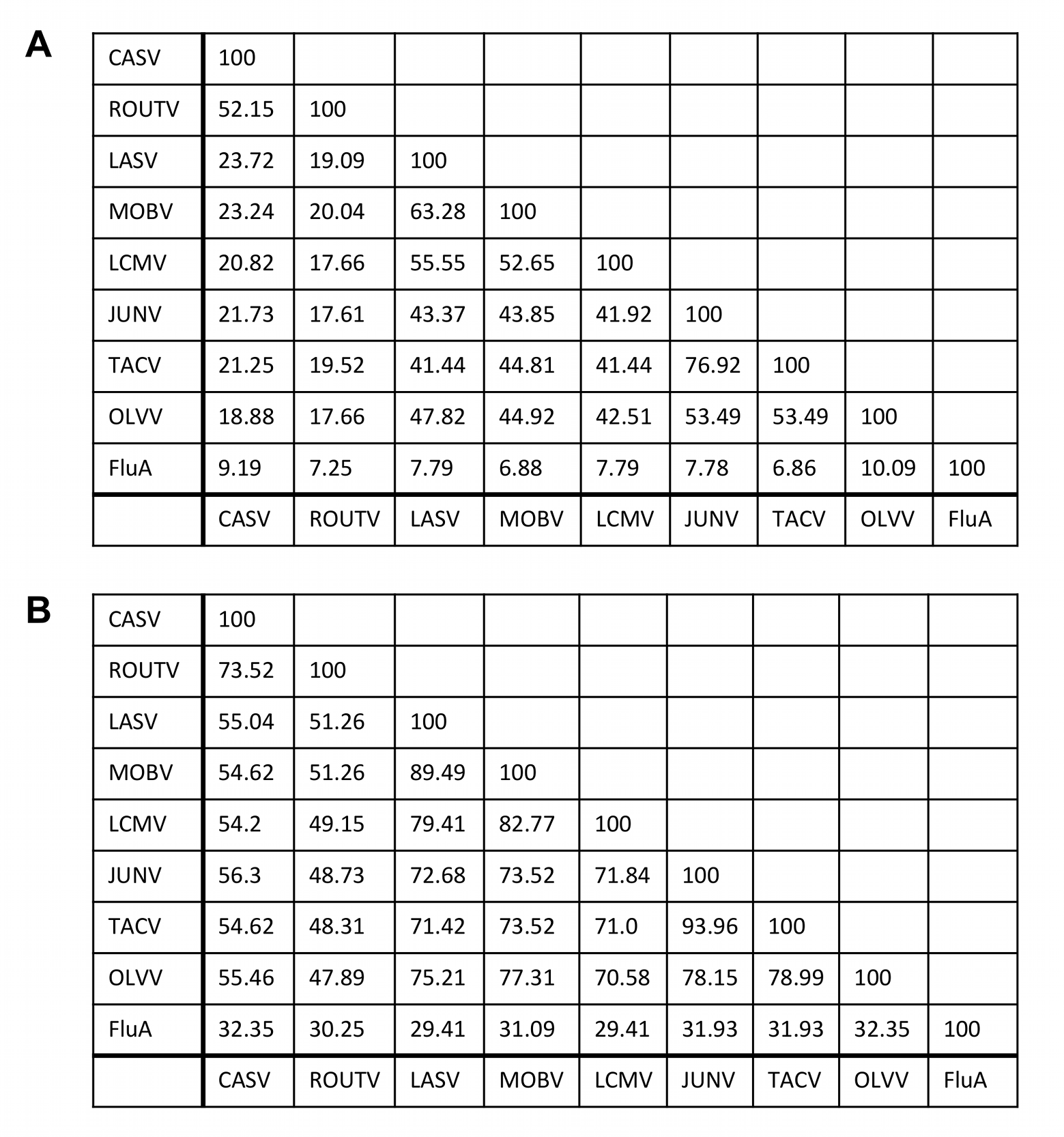

Supplement: S11 Fig — A) Identity matrix and B) similarity matrix of N-terminal sequences. Matrices were calculated based on the presented alignment of N-termini (S8 Fig) using the SIAS online tool (http://imed.med.ucm.es/Tools/sias.html) and values are given in percent relative to the mean length of sequences compared. Abbreviations: Full virus names are given in legend to S8 Fig. (TIF) [file ppat.1006400.s011.tif]

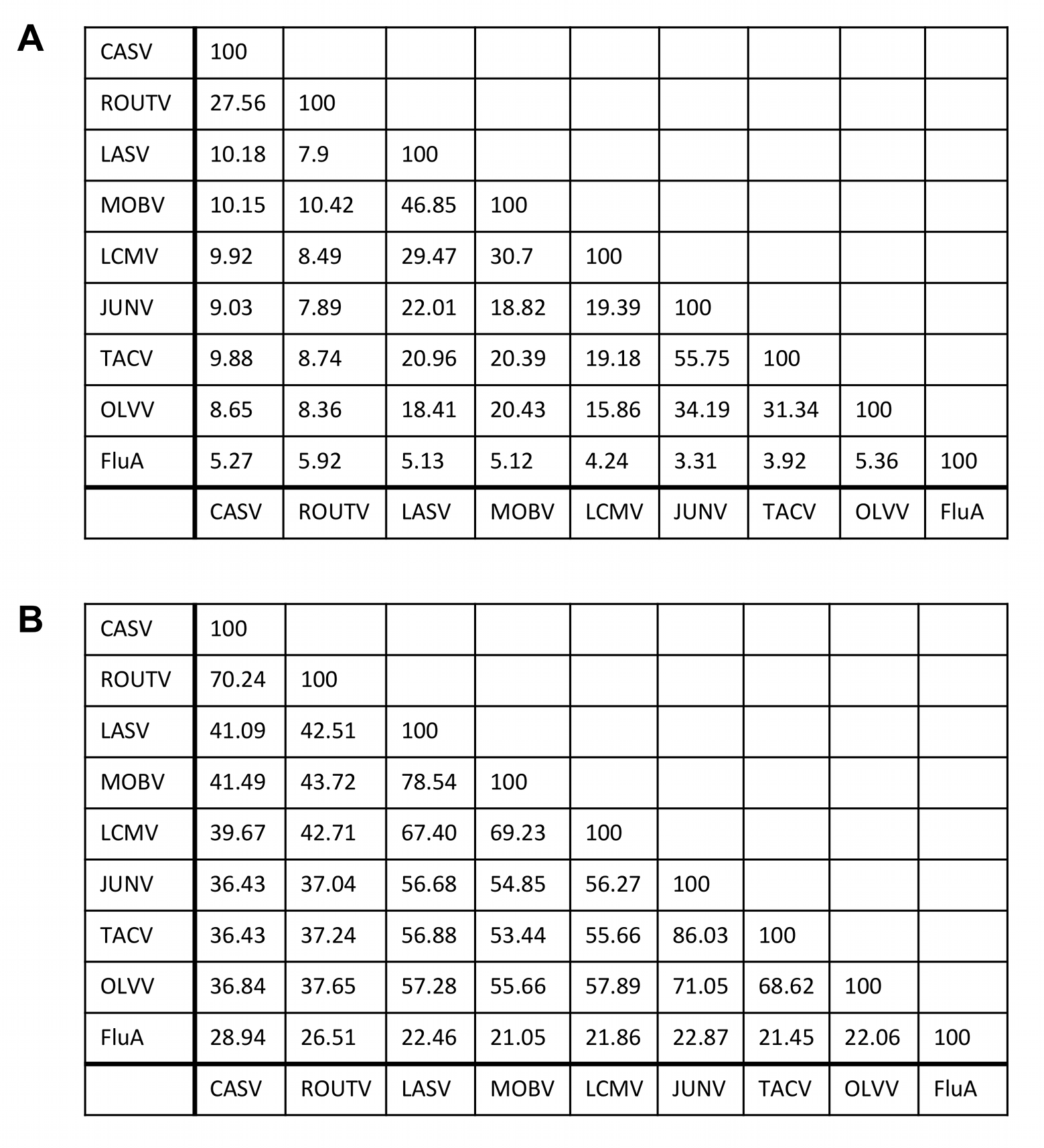

Supplement: S12 Fig — A) Identity matrix and B) similarity matrix of C-terminal sequences. Matrices were calculated based on the presented alignment of C-termini (S2 Fig) using the SIAS online tool (http://imed.med.ucm.es/Tools/sias.html) and values are given in percent relative to the mean length of sequences compared. Abbreviations: Full virus names are given in legend to S2 Fig. (TIF) [file ppat.1006400.s012.tif]

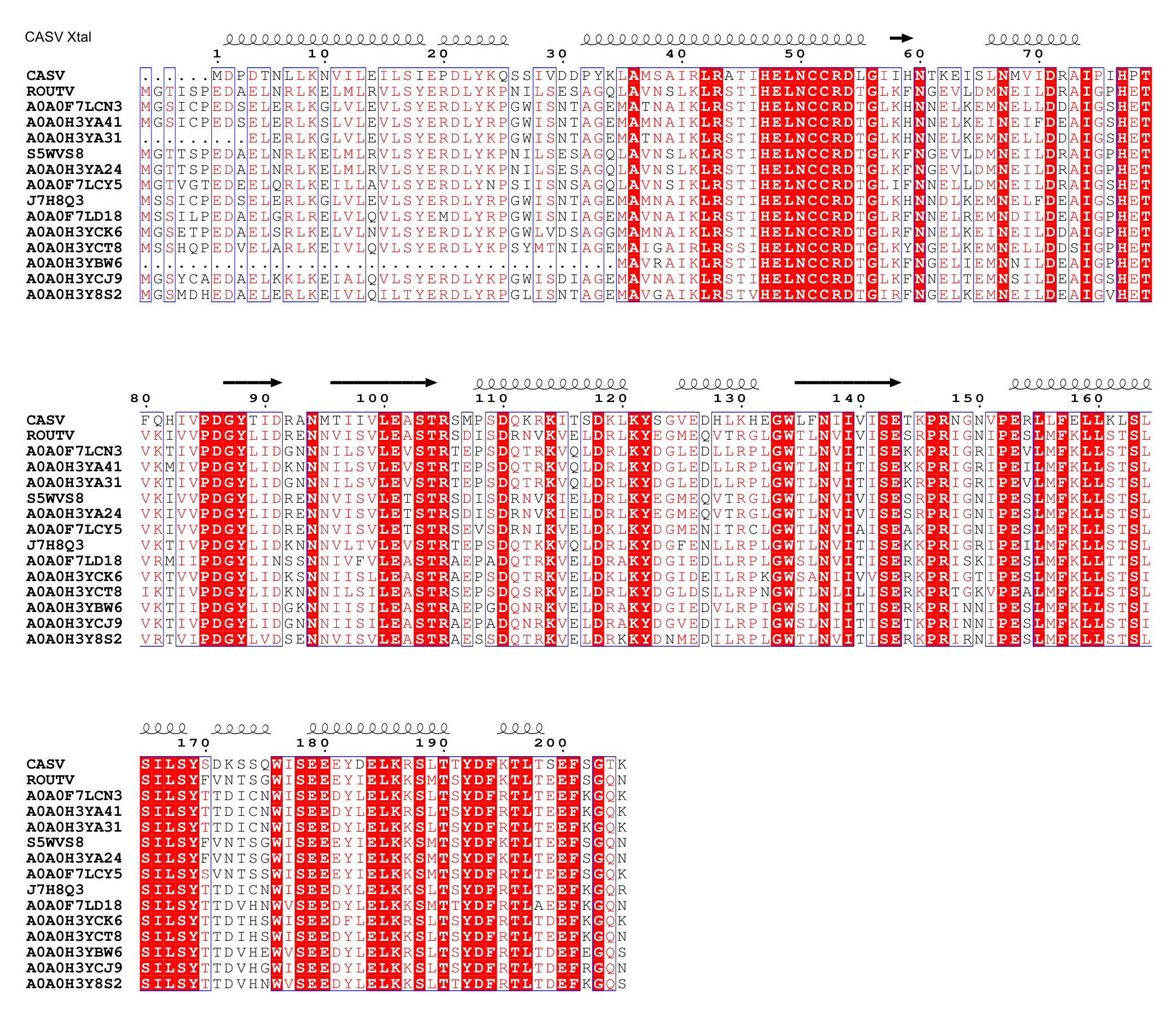

Supplement: S13 Fig — The alignment was generated using ClustalOmega [31] and includes sequences from L proteins of reptarenaviruses CASV (Uniprot-ID: J7HBG8), Boa arenavirus NL (ROUTV, M4PUV6) as well as 13 other reptarenavirus L protein sequences (Uniprot-IDs are given). The secondary structure of the CASV endonuclease crystal structure (CASV Xtal) is shown above the sequences. The alignment was drawn using the ESPript online tool (http://espript.ibcp.fr) [61]. (TIF) [file ppat.1006400.s013.tif]

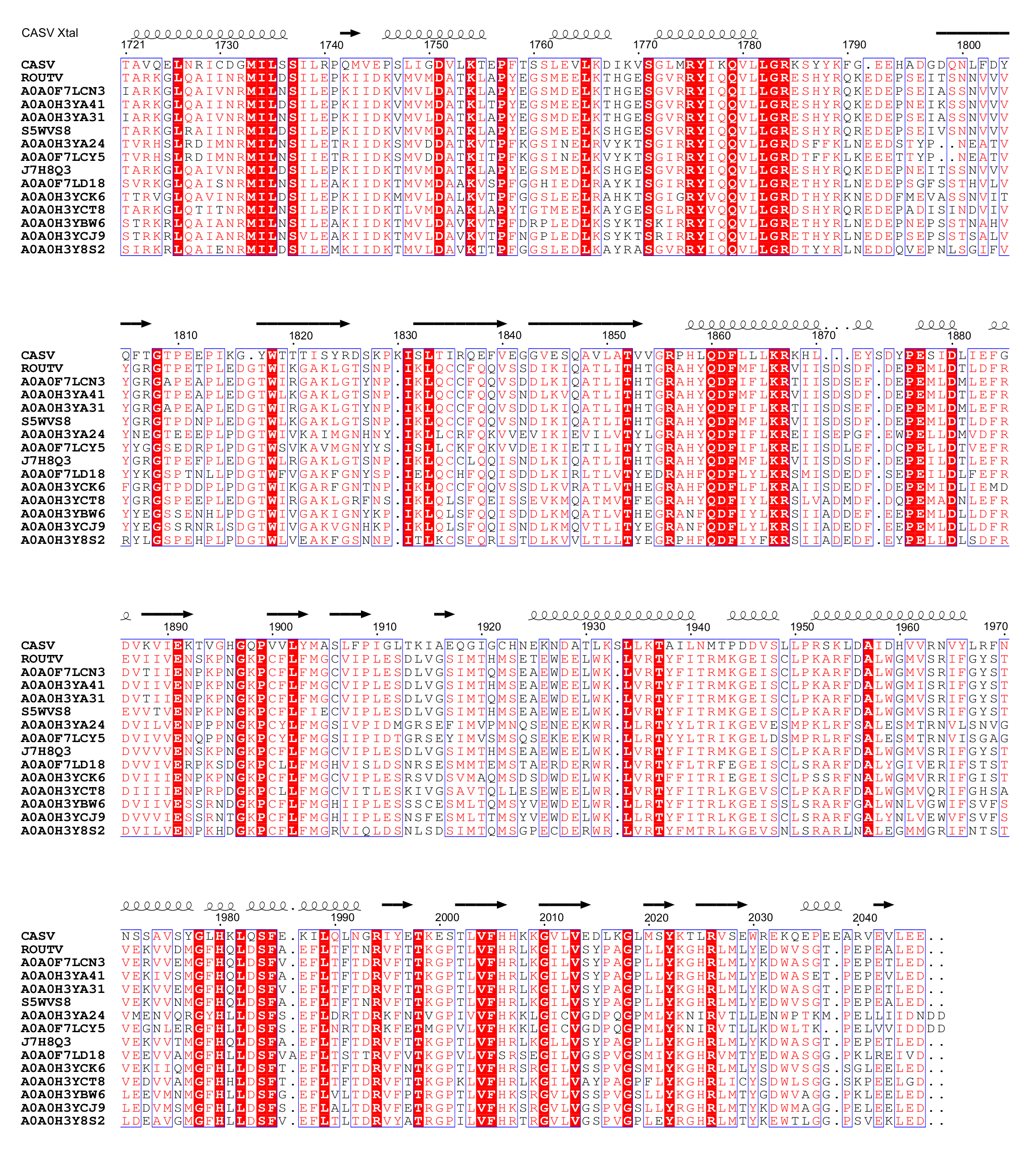

Supplement: S14 Fig — The alignment was generated using ClustalOmega [31] and includes sequences from L proteins of reptarenaviruses CASV (Uniprot-ID: J7HBG8), Boa arenavirus NL (ROUTV, M4PUV6) as well as 13 other reptarenavirus L protein sequences (Uniprot-IDs are given). The secondary structure of the CASV L-Cterm crystal structure (CASV Xtal) is shown above the sequences. The alignment was drawn using the ESPript online tool (http://espript.ibcp.fr) [61] with manual adjustments. (TIF) [file ppat.1006400.s014.tif]
