# Supplementary material for: Structural insights into reptarenavirus cap-snatching machinery
Source: PLoS Pathog. 2017 May 15;13(5):e1006400. doi: 10.1371/journal.ppat.1006400 (PMC5444859; doi:10.1371/journal.ppat.1006400)
Supplement: S1 Table — (DOC) [file ppat.1006400.s015.doc]

**Suppl Table S1. Crystallographic data and refinement statistics**

|  | **CASV L-Cterm_SeMet_** | **CASV L-Cterm_nativ_** | **CASV L-Cterm/ m^7^GTP** | **CASV L-Cterm domain 2** | **CASV Endo_SeMet_** | **CASV Endo_nativ_** |
| --- | --- | --- | --- | --- | --- | --- |
| **Data collection** |  |  |  |  |  |  |
| Synchrotron beamline | ESRF ID29 | ESRF ID29 | ESRF ID29 | PETRA P13 | PETRA P13 | PETRA P13 |
| Wavelength (Å) | 0.9788 | 0.9793 | 0.9793 | 0.9762 | 0.9762 | 0.9762 |
| Resolution (Å) | 49-2.4 | 63.95-2.009 (2.081-2.009) | 63.93-1.99 (2.061-1.99) | 64.48-1.776 (1.84-1.776) | 75.57-2.365 (2.45-2.365) | 90.73-1.931 (2-1.931) |
| Space group | P2_1_2_1_2_1_ | P2_1_2_1_2_1_ | P2_1_2_1_2_1_ | C121 | P4_3_2_1_2 | P2_1_2_1_2_1_ |
| Cell dimensions |  |  |  |  |  |  |
| a, b, c (Å) | 75.87,  76.82,  117.289 | 76.3917, 76.9421, 116.92 | 76.3396, 76.6012, 116.06 | 74.83, 42.87, 70.83 | 96.5019, 96.5019, 243.019 | 56.9698, 112.342, 153.848 |
| α, β, γ (°) | 90, 90, 90 | 90, 90, 90 | 90, 90, 90 | 90, 114.444, 90 | 90, 90, 90 | 90, 90, 90 |
| Total reflections | 344293 (28360) | 199980 (15135) | 132563 (9708) | 74503 (4420) | 1098724 (103754) | 857759 (53485) |
| Unique reflections | 51627 (5812) | 45998 (3343) | 46340 (3259) | 18559 (1062) | 47978 (4601) | 75005 (4408) |
| Multiplicity | 6.7 (4.9) | 4.3 (4.5) | 2.9 (3.0) | 4.0 (4.2) | 22.9 (22.6) | 11.4 (11.9) |
| Completeness (%) | 0.99 (0.97) | 0.99 (0.99) | 0.98 (0.99) | 0.97 (0.98) | 1.00 (1.00) | 1.00 (1.00) |
| Mean I/sigma(I) | 11.6 (1.4) | 8.1 (1.5) | 4.3 (0.8) | 10.0 (1.6) | 8.6 (3.1) | 14.9 (4.8) |
| Wilson B-factor | 48.22 | 31.49 | 25.26 | 35.05 | 23.04 | 22.76 |
| R-merge | 0.108 (0.99) | 0.04953 (0.4792) | 0.1481 (1.204) | 0.02015 (0.2814) | 0.07159 (0.228) | 0.02479 (0.1382) |
| R-meas | 0.117 (1.12) | 0.07005 (0.6777) | 0.2094 (1.702) | 0.02849 (0.398) | 0.1012 (0.3224) | 0.03505 (0.1955) |
| **Refinement** |  |  |  |  |  |  |
| Resolution (Å) |  | 64-2.01 | 64-1.99 | 64-1.78 |  | 90.8-1.93 |
| Reflections used in refinement |  | 45931 (4524) | 45836 (4389) | 19333 (1916) |  | 74918 (7396) |
| Reflections used for R-free |  | 2320 (232) | 2326 (219) | 992 (122) |  | 3753 (367) |
| R-work |  | 0.1977 (0.3087) | 0.2225 (0.3591) | 0.2139 (0.3358) |  | 0.1731 (0.2074) |
| R-free |  | 0.2421 (0.3495) | 0.2760 (0.3892) | 0.2770 (0.3771) |  | 0.2126 (0.2776) |
| No. of atoms |  |  |  |  |  |  |
| protein |  | 5247 | 5264 | 1560 |  | 6562 |
| ligand/ ion |  | 19 | 33 |  |  | 20 |
| Water molecules |  | 401 | 474 | 94 |  | 620 |
| Average B-factors (Å^2^) |  |  |  |  |  |  |
| protein |  | 38.62 | 31.62 | 47.49 |  | 26.31 |
| ligand |  | 38.20 | 59.87 |  |  | 29.08 |
| solvent |  | 44.20 | 35.98 | 54.58 |  | 34.42 |
| R.m.s deviations |  |  |  |  |  |  |
| bond lengths (Å) |  | 0.008 | 0.008 | 0.008 |  | 0.009 |
| bond angles (°) |  | 0.93 | 0.93 | 1.000 |  | 1.07 |
| Ramachandran (%) |  |  |  |  |  |  |
| favored |  | 98 | 96 | 97 |  | 100 |
| allowed |  | 2 | 3.5 | 3 |  | 0.37 |
| outliers |  | 0.15 | 0.15 | 0 |  | 0 |
| Rotamer outliers (%) |  | 0.84 | 0.5 | 0 |  | 1.2 |
| **PDB code** |  | **5MUS** | **5MUY** | **5MUZ** |  | **5MV0** |
