# Supplementary material for: Structural insights into reptarenavirus cap-snatching machinery
Source: PLoS Pathog. 2017 May 15;13(5):e1006400. doi: 10.1371/journal.ppat.1006400 (PMC5444859; doi:10.1371/journal.ppat.1006400)
Supplement: S2 Table — (DOC) [file ppat.1006400.s016.doc]

**Suppl Table S2. Analysis of L protein mutants in Lassa virus replicon system.**

| Mutant^1^ | Renilla luciferase activity (sRLU)  ––––––––––––––––––––––––––––––––– | | | | | | RNA expression level (Northern blot signal)  ––––––––––––––––––––––––––––––––––– | |
| --- | --- | --- | --- | --- | --- | --- | --- | --- |
|  | % of wild-type^2^ | | | Signal-to-noise ratio^3^ | | | Antigenome level, % of wild-type^4^ | mRNA-to-antigenome ratio, relative to wild-type^5^ |
| W1915L | 36.34 | +/- | 4.09 | 54.6 | +/- | 2.8 | 255.39 | 0.44 |
| W1915N | 39.68 | +/- | 9.96 | 59.7 | +/- | 6.8 | 137.48 | 0.48 |
| **W1915E** | **31.07** | **+/-** | **6.78** | **46.7** | **+/-** | **4.6** | **291.22** | **0.28** |
| W1915K | 57.28 | +/- | 11.53 | 86.1 | +/- | 7.8 | 272.43 | 0.53 |
| W1915F | 95.62 | +/- | 10.59 | 143.8 | +/- | 7.2 | 216.26 | 0.64 |
| F1985L | 120.16 | +/- | 19.72 | 180.7 | +/- | 13.4 | 164.71 | 1.06 |
| F1985D | 0.63 | +/- | 1.86 | 1.0 | +/- | 1.3 | 15.01 | 1.29 |
| F1985K | 0.75 | +/- | 2.60 | 1.1 | +/- | 1.8 | 16.31 | 1.26 |
| F1985Y | 122.56 | +/- | 11.83 | 184.3 | +/- | 8.0 | 143.44 | 0.81 |
| Y2004L | 34.93 | +/- | 12.57 | 52.5 | +/- | 8.5 | 178.49 | 0.46 |
| Y2004F | 86.67 | +/- | 27.13 | 130.3 | +/- | 18.4 | 113.44 | 0.98 |
| V2034A | 88.18 | +/- | 23.59 | 132.6 | +/- | 16.0 | 282.14 | 0.84 |
| V2034S | 12.58 | +/- | 9.46 | 18.9 | +/- | 6.4 | 77.99 | 0.84 |
| V2034D | 0.90 | +/- | 1.61 | 1.4 | +/- | 1.1 | 60.10 | 0.40 |
| **E2041L** | **45.85** | **+/-** | **4.57** | **68.9** | **+/-** | **3.1** | **274.15** | **0.32** |
| E2041D | 94.30 | +/- | 17.56 | 141.8 | +/- | 11.9 | 300.17 | 0.59 |
| **E2041K** | **16.09** | **+/-** | **3.75** | **24.2** | **+/-** | **2.5** | **251.07** | **0.28** |
| F2042L | 112.81 | +/- | 9.14 | 169.6 | +/- | 6.2 | 146.52 | 0.79 |
| F2042S | 112.80 | +/- | 7.75 | 169.6 | +/- | 5.3 | 162.14 | 0.81 |
| **F2042D** | **15.05** | **+/-** | **0.19** | **22.6** | **+/-** | **0.1** | **235.16** | **0.26** |
| F2042W | 104.84 | +/- | 26.93 | 157.6 | +/- | 18.3 | 174.58 | 0.65 |
| F2042Y | 121.64 | +/- | 22.46 | 182.9 | +/- | 15.2 | 163.92 | 0.61 |

^1^ Mutants with selective defect in mRNA synthesis are shown in boldface.

^2^ Standardized relative light unit (sRLU) value (wild-type = 100%). Mean of ≥3 independent transfection experiments.

^3^ sRLU value of mutant divided by sRLU value of negative control mutant containing a mutation in the catalytic site of the RNA-dependent RNA polymerase. Mean of ≥3 independent transfection experiments.

^4^ Antigenome signals in Northern blots were quantified via intensity profiles using ImageJ software (Schneider, C. A.; Rasband, W. S. & Eliceiri, K. W. (2012), "NIH Image to ImageJ: 25 years of image analysis", Nature methods 9(7): 671-675) (wild-type = 100%).

^5^ RNA signals in Northern blots were quantified and the mRNA-to-antigenome signal ratio was calculated. The wild-type ratio was set at 1 for each experiment (i.e. the signal ratio of a mutant was normalized with the wild-type ratio) to render independent experiments comparable. Examination of the intensity profiles revealed residual signals at the mRNA position (about 10–30% of the wild-type mRNA-to-antigenome signal ratio) for mutants negative in the Ren-Luc assay. Therefore, these signals do not correspond to functional mRNA, but may be prematurely terminated antigenome.
