# Supplementary material for: Structural insights into reptarenavirus cap-snatching machinery
Source: PLoS Pathog. 2017 May 15;13(5):e1006400. doi: 10.1371/journal.ppat.1006400 (PMC5444859; doi:10.1371/journal.ppat.1006400)
Supplement: S3 Table — This list contains tested fragments that were either insoluble, not suitable for crystallization trials or could not be crystallized successfully. (DOC) [file ppat.1006400.s017.doc]

**Suppl Table S3. List of tested L protein C-term fragments**

This list contains tested fragments that were either insoluble, not suitable for crystallization trials or could not be crystallized successfully.

| **Virus** | **N terminus** | **C terminus** | **Solubility in *E. coli**** |  | **Virus** | **N terminus** | **C terminus** | **Solubility in *E. coli**** |
| --- | --- | --- | --- | --- | --- | --- | --- | --- |
| LASV AV  Q6Y630 | 1729 | 2220 | - |  | CASV  J7HBG8 | 1693 | 2046 | + |
|  | 1729 | 1952 | - |  |  | 1721 | 2046 | ++ |
|  | 1742 | 2220 | - |  |  | 1743 | 2046 | - |
|  | 1754 | 2220 | - |  |  | 1760 | 2046 | - |
|  | 1772 | 2220 | - |  |  | 1785 | 2046 | + |
|  | 1792 | 2220 | - |  | Chapare virus  B2C4J3 | 1798 | 2208 | - |
|  | 1806 | 2220 | - |  |  | 1826 | 2208 | - |
|  | 1823 | 2220 | + |  |  | 1846 | 2208 | - |
|  | 1823 | 2206 | + |  |  | 1875 | 2208 | - |
|  | 1838 | 2220 | ++ |  |  | 1900 | 2208 | - |
|  | 1855 | 2220 | - |  | Guanarito virus  Q6UY70 | 1790 | 2198 | - |
|  | 1886 | 2220 | - |  |  | 1818 | 2198 | - |
|  | 1912 | 2220 | + |  |  | 1867 | 2198 | - |
|  | 1912 | 2212 | + |  |  | 1892 | 2198 | - |
|  | 1912 | 2206 | + |  | Luna virus  G3XEV9 | 1803 | 2215 | - |
|  | 1934 | 2220 | - |  |  | 1830 | 2215 | + |
|  | 1953 | 2220 | - |  |  | 1851 | 2215 | - |
|  | 1971 | 2220 | - |  |  | 1881 | 2215 | - |
|  | 2006 | 2220 | - |  |  | 1906 | 2215 | + |
|  | 2028 | 2220 | - |  | Pichinde virus  Q915A5 | 1775 | 2190 | - |
| LASV Bantou 289 | 1835 | 2217 | + |  |  | 1802 | 2190 | - |
| MOPV  G3LUX0 | 1744 | 2237 | - |  |  | 1822 | 2190 | - |
|  | 1744 | 1966 | - |  |  | 1851 | 2190 | - |
|  | 1756 | 2237 | - |  |  | 1876 | 2190 | - |
|  | 1768 | 2237 | - |  | ROUTV  M4PUV6 | 1712 | 2065 | - |
|  | 1786 | 2237 | - |  |  | 1741 | 2065 | ++ |
|  | 1806 | 2237 | - |  |  | 1765 | 2065 | - |
|  | 1821 | 2237 | - |  |  | 1796 | 2065 | - |
|  | 1837 | 2237 | - |  |  | 1821 | 2065 | - |
|  | 1869 | 2237 | - |  | Sabia virus  Q6UY61 | 1802 | 2212 | - |
|  | 1900 | 2237 | - |  |  | 1830 | 2212 | - |
|  | 1926 | 2237 | - |  |  | 1850 | 2212 | - |
|  | 1948 | 2237 | - |  |  | 1879 | 2212 | - |
|  | 1971 | 2237 | - |  |  | 1904 | 2212 | - |
|  | 1986 | 2237 | - |  | Tamiami virus  A9JR23 | 1800 | 2221 | - |
|  | 2021 | 2237 | - |  |  | 1828 | 2221 | - |
| LCMV  P14240 | 1722 | 2210 | - |  |  | 1848 | 2221 | - |
|  | 1722 | 1944 | - |  |  | 1877 | 2221 | - |
|  | 1815 | 2210 | - |  |  | 1902 | 2221 | - |
|  | 1847 | 2210 | - |  |  |  |  |  |
|  | 1878 | 2210 | - |  |  |  |  |  |
|  | 1903 | 2210 | - |  |  |  |  |  |
|  | 1926 | 2210 | - |  |  |  |  |  |
|  | 1949 | 2210 | - |  |  |  |  |  |
|  | 1964 | 2210 | - |  |  |  |  |  |
|  | 1999 | 2210 | - |  |  |  |  |  |

* Solubility of the resulting protein fragment expressed in *E. coli* was rated as 'insoluble' (-), 'soluble' (+) or 'highly soluble' (++)
