# Supplementary material for: Structural insights into reptarenavirus cap-snatching machinery
Source: PLoS Pathog. 2017 May 15;13(5):e1006400. doi: 10.1371/journal.ppat.1006400 (PMC5444859; doi:10.1371/journal.ppat.1006400)
Supplement: S1 Methods — The methods of LASV minireplicon system and the endonuclease assay are described in detail. (DOC) [file ppat.1006400.s018.doc]

**Supplementary Methods**

**Testing of L protein mutants in the LASV minireplicon system**

The experiments were performed in the context of the T7 RNA polymerase-based Lassa virus minireplicon system essentially as described previously [1-3]. L gene mutants were generated by mutagenic PCR using pCITE-L as a template. The PCR products containing the functional cassette for expression of mutant L protein were purified, quantified spectrophotometrically, and used for transfection without prior cloning. The presence of the artificial mutation was ascertained by sequencing. BSR-T7/5 cells stably expressing T7 RNA polymerase [4] were transfected per well of a 24-well plate with 250 ng of minigenome expressing Renilla luciferase (Ren-Luc), 250 ng of L gene PCR product , 250 ng of pCITE-NP expressing NP, and 10 ng of pCITE-FF-luc expressing firefly luciferase as an internal transfection control. One day after transfection, total RNA was purified using an RNeasy Mini Kit (Qiagen) for Northern blotting or cells were lysed in 100 µl of passive lysis buffer (Promega) per well, and assayed for firefly luciferase and Ren-Luc activity using the dual-luciferase reporter assay system (Promega). Ren-Luc levels were corrected with the firefly luciferase levels (resulting in standardized relative light units [sRLU]) to compensate for differences in transfection efficiency or cell density.

For Northern blot analysis, 500 ng of RNA was separated in a 1.5%-agarose–formaldehyde gel and transferred onto a Hybond N^+^ membrane (Amersham Pharmacia Biotech). Blots were hybridized with a ^32^P-labeled riboprobe targeting the Ren-Luc gene, and RNA bands were visualized by autoradiography using a Typhoon scanner (GE Healthcare).

To verify expression of L protein mutants, BSR-T7/5 cells in a well of a 24-well were transfected with 500 ng of PCR product expressing L protein mutants tagged at C-terminus with a 3xFLAG sequence. To enhance the expression level, the cells were additionally inoculated with modified vaccinia virus Ankara expressing T7 RNA polymerase (MVA-T7) [5]. Cytoplasmic lysate was separated in a 3–8% Tris-acetate polyacrylamide gel, transferred to nitrocellulose membrane (Schleicher & Schuell), and detected by immunoblotting using peroxidase-conjugated anti-FLAG M2 antibody (1:10,000) (A8592; Sigma-Aldrich). L protein bands were visualized by chemiluminescence using SuperSignal West Femto substrate (Pierce) and a FUSION SL image acquisition system (Vilber Lourmat).

**Endonuclease assay**

The experiments were performed essentially as described previously [6]. Endonuclease activity was measured by incubating 1 μM protein with 0.1 μM ^32^P-labeled single stranded 27mer or 40mer polyA RNA substrate. Reaction conditions contained 50 mMTris, pH 7.3, 250mM NaCl, 5% glycerol, 0.25 U/μl RNasin (Promega) and either no MnCl_2_ or 5 mM MnCl_2_. Reactions were carried out at 37°C for 1 h. The reaction was stopped by adding 2 x loading buffer (95% formamide, 18 mM ethylenediaminetetraacetic acid (EDTA), 0.025% sodium dodecyl sulfate, xylene cyanol, and bromophenol blue) and heating the samples to 98°C for 5 min. The reaction products were separated by 8 M urea, 20% polyacrylamide, Tris-borate-EDTA gel electrophoresis and visualized by phosphor screen autoradiography using a Typhoon scanner (GE Healthcare).

1. Hass M, Golnitz U, Muller S, Becker-Ziaja B, Gunther S. Replicon system for Lassa virus. Journal of Virology. 2004;78(24):13793-803.

2. Hass M, Lelke M, Busch C, Becker-Ziaja B, Gunther S. Mutational evidence for a structural model of the Lassa virus RNA polymerase domain and identification of two residues, Gly1394 and Asp1395, that are critical for transcription but not replication of the genome. Journal of Virology. 2008;82(20):10207-17.

3. Morin B, Coutard B, Lelke M, Ferron F, Kerber R, Jamal S, et al. The N-terminal domain of the arenavirus L protein is an RNA endonuclease essential in mRNA transcription. PLoS Pathog. 2010;6(9):e1001038.

4. Buchholz UJ, Finke S, Conzelmann KK. Generation of bovine respiratory syncytial virus (BRSV) from cDNA: BRSV NS2 is not essential for virus replication in tissue culture, and the human RSV leader region acts as a functional BRSV genome promoter. J Virol. 1999;73(1):251-9.

5. Sutter G, Ohlmann M, Erfle V. Non-replicating vaccinia vector efficiently expresses bacteriophage T7 RNA polymerase. FEBS Lett. 1995;371(1):9-12.

6. Fernandez-Garcia Y, Reguera J, Busch C, Witte G, Sanchez-Ramos O, Betzel C, et al. Atomic Structure and Biochemical Characterization of an RNA Endonuclease in the N Terminus of Andes Virus L Protein. PLoS Pathog. 2016;12(6):e1005635.
